# The impact of tax burden on welfare attitudes: Micro evidence from welfare states

**DOI:** 10.1371/journal.pone.0311047

**Published:** 2024-11-18

**Authors:** Yinan Guo, Ting Gong

**Affiliations:** 1 Institute of Guangdong Hong Kong and Macao Development Studies, Sun Yat-Sen University, Guangzhou, Guangdong, China; 2 School of Law, Sun Yat-Sen University, Guangzhou, Guangdong, China; Kırklareli University: Kirklareli Universitesi, TÜRKIYE

## Abstract

The attitudes of the general public with regard to social welfare are of crucial importance in determining the efficacy and stability of a nation’s welfare system. The manner in which taxation is employed as a means of funding mechanism for welfare policies is of great consequence. Nevertheless, existing research on the subject of welfare attitudes has largely neglected the tax perspective, underscoring the need for investigations that bridge this gap and provide a more comprehensive understanding of the intertwined dynamics between taxation and public perception of social benefits. This study investigates the influence of tax burdens on attitudes towards welfare using an ordered probit model applied to data from the 2019 International Social Survey Program (ISSP 2019 Social Inequality V), encompassing 11 welfare states. Our key findings are as follows: (1) Empirical analysis reveals that a moderate tax burden correlates with a reduction in public expectations regarding governmental responsibility for welfare provision. (2) Heterogeneity analysis elucidates a negative association between tax burdens and welfare attitudes across diverse welfare regimes. (3) The mediating effect test suggests that perceptions of social fairness partially mediate the relationship between tax burdens and welfare attitudes. (4) The moderating effect test indicates that government efficacy negatively moderates the impact of tax burden on welfare attitudes. This study offers insightful perspectives for policymakers aiming to design and implement tax systems that align effectively with societal structures.

## 1 Introduction

In the landscape of modern governance, the provision of welfare by governments stands as a cornerstone of societal progress. Its significance is profound, fostering economic growth, mitigating inequality, and ensuring social equilibrium [1–5]. Since the mid-20th century, industrialized nations embarked on a journey towards welfare statehood, culminating in comprehensive systems designed to safeguard citizens’ well-being. However, the latter half of the 20th century presented formidable challenges to these welfare paradigms, as globalization, economic downturns, fiscal crises, and shifting social dynamics tested their resilience, prompting debates on sustainability and evolution [6–9]. Scholars, like Pierson [10], proposed nuanced strategies to recalibrate welfare systems, acknowledging the imperative of expanding social safety nets while curbing expenditure. Despite enduring strains, welfare states persist, fueled by entrenched institutional norms and a growing public demand for social security. Yet, the erosion of post-war welfare consensus and legitimacy crises underscore the pivotal role of public attitudes towards welfare in shaping its trajectory [11].

Consequently, scholarly inquiries into welfare attitudes burgeoned, seeking to comprehend citizens’ perceptions of state responsibility through the lens of goals, processes, and outcomes of welfare provision. Scholarship on welfare attitudes, rooted in diverse disciplines, has scrutinized the interplay of individual and contextual factors shaping welfare sentiments [12–15]. In accordance with the rational man hypothesis, it is believed that individuals’ specific behaviors are driven by their own interests, and their welfare attitude preferences are influenced by benefits and losses and individual socioeconomic status, and the public tends to support government actions and government responsibilities that can directly benefit them, and believe that the government should take more responsibility for welfare services [16]. Simultaneously, an individual’s welfare attitudes are influenced by various factors, including gender, age, marital status, education level, income level, and occupation. Individual characteristics also shape perceptions formed during socialization [17, 18]. Regarding gender, women may hold more positive attitudes towards welfare due to their relative disadvantage in the job market compared to men, as well as their greater caregiving responsibilities in the household and higher likelihood of benefiting from various welfare policies [19–21]. There is no consensus among scholars on the effect of education on welfare attitudes. One group of views suggests that educational attainment is generally proportional to income level. Higher educational attainment tends to be associated with higher income levels, a belief in the importance of earning social benefits through personal endeavor, and less dependence on social benefits. This leads to correspondingly lower claims on the state’s responsibility for welfare [22, 23]; yet another category of research suggests that the more educated people are, the stronger their sense of fairness and justice, the more they tend to identify with the concept of social equality, and the more they tend to support government responsibility for welfare [24]. Empirical studies for European countries have shown a negative correlation between income and welfare attitudes, with income being the strongest predictor [25–27]. Scholars suggest that welfare attitudes cannot be adequately explained by a single factor. Instead, more detailed combinations of variables, such as immigrant background, political inclination, and subjective feelings of belonging to a class, should be proposed for analysis. Welfare attitudes are inversely proportional to the social resources and risk tolerance available to individuals. Individuals with a high ability to collect social resources and a high tolerance for risk are more likely to believe that individuals should be responsible for their own welfare and that they should be remunerated by obtaining rewards in the market rather than relying on the government to provide welfare. Therefore, individuals who are male, middle-aged, or have a high income are more likely to identify with economic individualism. Conversely, women, the elderly, and those with low incomes are more likely to identify with welfare state policies [28–32].

Meanwhile, some scholars argue that the self-interest hypothesis alone cannot fully explain the variations in individual attitudes towards welfare. A more comprehensive understanding necessitates research into a broader spectrum of social values, cultural dynamics, and institutional frameworks supporting welfare systems. Welfare attitudes are shaped by diverse ethnic cultures, values, and welfare systems, which interpret fairness and justice, ultimately influencing people’s perceptions and support for government distribution of welfare among different societal members [33]. Citizens in a welfare state typically share a common historical background, social traditions, culture, and values. These perceptions implicitly shape welfare attitudes and affect the level of support for the government’s role in welfare provision. The concept of welfare culture plays a significant role in shaping social welfare systems and is a crucial factor in explaining the variations observed across different welfare systems [19, 34–36]. With the process of modernization, societies have become more affluent, leading people to shift their focus from material, physical, and safety concerns to self-realization and community solidarity, reflecting post-materialist values [37–39]. If economic benefits, political rights, development opportunities, and social welfare are equitably distributed among members of society within a country, the principle of justice is stronger. Citizens’ value orientations and judgments of the principle of justice significantly affect their attitudes toward government welfare provision. The public’s recognition of justice and equality as a social value leads to a positive attitude toward welfare policies. However, they may disapprove of potential negative consequences. Compared to Western welfare states, East Asian welfare states (regions) are deeply influenced by Confucian culture, which emphasizes strong family-oriented concepts and individual family welfare responsibility. This results in a tendency to reduce the government’s involvement in the field of social welfare [40–42].

Yet, scant attention has been paid to the nexus between taxation and welfare attitudes [43]. Taxation emerges as a linchpin in this discourse, serving as a tool for macroeconomic regulation and wealth redistribution [44, 45]. As advocated by Hobbes in Leviathan, equitable taxation is integral to achieving maximal social welfare by rationalizing wealth distribution. The confluence of burgeoning tax burdens and lofty welfare aspirations engenders discontent, fueling debates on tax justice and welfare efficacy [46–48]. Striking a balance between funding welfare provisions and alleviating tax pressures becomes a precarious task, fraught with political and economic complexities [49, 50]. Public resistance to tax hikes underscores concerns over fairness and the equitable distribution of welfare benefits [51, 52]. Excessive taxation risks exacerbating social tensions and undermining public trust in governmental welfare initiatives [53–56]. By contrast, if taxation remains insufficient, it may result in inadequate funding for essential welfare programs, thereby exacerbating social inequalities and hindering efforts to address systemic issues of poverty and inequality. Thus, understanding the intricate relationship between taxation and welfare attitudes is imperative for formulating effective and sustainable welfare policies.

Given the pivotal role of taxation in financing welfare states and its implications for social equity, addressing the research gap concerning the nexus between tax burdens and welfare attitudes is imperative. Thus, this study aims to empirically investigate the impact of tax burdens on welfare attitudes, utilizing data from the 2019 International Social Survey Programme (ISSP 2019) on social inequality. Specifically, we seek to ascertain how variations in tax burdens influence individuals’ perceptions regarding whether the state and government should bear more or less responsibility in providing welfare. Additionally, by examining multiple welfare states with diverse tax systems, we endeavor to elucidate the mediating role of social equity perceptions in the relationship between tax burdens and welfare attitudes. Our analysis not only enhances theoretical understanding of welfare attitudes but also provides policymakers with practical insights for addressing the challenging task of reconciling tax burdens with welfare aspirations.

## 2 Theoretical analysis and research hypothesis

### 2.1 Conceptual definition

#### Welfare attitudes

The concept of welfare attitudes is multidimensional, it refers to the views, opinions, and beliefs formed by the public about the behavior and policies of pluralistic welfare-providing entities, mainly the government. It can also include public attitudes toward government welfare policies and the distribution and redistribution of resources and life opportunities [23].

#### Tax burden

It refers to the economic costs or burdens that taxes impose on economic agents, such as individuals and businesses. The distribution of the tax burden involves the proportion of burden sharing among different economic agents, which determines the fairness of taxation [57, 58]. This study discusses the subjective perception of the tax burden by individuals, rather than their actual tax burden.

#### Perceived fairness

The concept of perceived fairness involves an individual’s perception of the fairness and equality of the distribution of social resources. It is a complex concept that includes factors such as social justice, social comparison, social culture, and individual values [59].

#### Government efficacy

It refers to the ability of a government to effectively formulate and implement sound policies, deliver public services efficiently, and maintain a stable and transparent administration. It encompasses aspects such as administrative competence, regulatory quality, and the absence of corruption, which together determine how well a government can achieve its goals and meet the needs of its citizens.

### 2.2 Tax burden and welfare attitudes

According to the classical theory of tax price, taxes are essentially an exchange based on the market economy and the concept of social contract, as the price paid by citizens to obtain public goods provided by the government [60]. Tax Price Theory posits that taxes function as a transaction within a market economy and a social contract, wherein citizens pay for access to public goods provided by the government. Taxation is a tool to enhance the common well-being of the state and the people, provided that the taxpayers are relatively satisfied with the social welfare. Based on the social contract, the government undertakes the function of providing public goods on behalf of the people, and the government needs to bear the corresponding costs of providing public goods. Through taxation, members of the community pay taxes to transfer social resources from the private sector to the public sector to compensate for the costs of providing public goods and public services [61–63]. Analogous to purchasing individual commodities, taxation establishes an exchange relationship where monetary contributions correspond to the value of the benefits acquired, reflecting the equivalence principle in the exchange of goods. While taxation is obligatory and non-reciprocal—empowering the government to extract benefits from taxpayers without direct compensation—it mirrors the exchange of goods in market transactions, where the price reflects the value of the goods exchanged. Consequently, when public goods fail to meet societal expectations, individuals may perceive the tax burden as unjustifiable, prompting calls for increased government investment in welfare or enhanced redistribution of social benefits [64, 65].

The concept of fiscal illusion, articulated by Poviani and Buchanan, reveals the intricate connection between perceptions of taxation and attitudes towards welfare, highlighting a complex interplay that extends beyond economic transactions. This concept emphasizes how governments, pursuing greater fiscal authority and increased spending, inadvertently obscure the actual tax burden on individuals, thus distorting the fiscal-social contract [66, 67]. Taxpayers frequently underestimate their actual tax obligations, which leads to a distorted comprehension of the intricate relationship between taxation and the benefits derived from public goods and services [68]. The discrepancy between perceived and actual tax burdens lies at the core of this issue. Despite socio-economic progress, individuals from all societal strata frequently fail to accurately assess the costs associated with accessing public services. This discrepancy fosters the perception that the tax burden is less onerous than it actually is, blurring the distinction between taxes paid and benefits received. The perceived tax burden, which is often only a fraction of the actual economic cost provided by the government or possibly exceeding it, fails to accurately reflect the financial burden borne by taxpayers. Therefore, this discourse examines the perceived tax burden of individuals, emphasizing its divergence from the actual tax burden [69]. We recognise the existence of fiscal illusion and its influence on public psychology and socio-economic factors. Furthermore, we examine the relationship between tax burdens and societal attitudes towards welfare in the context of fiscal illusion. In essence, the entanglement of fiscal illusion, taxation, and welfare attitudes exemplifies a multifaceted socio-economic phenomenon, reflective of the intricate dynamics between governance, economics, and human behavior. The distortion of perceptions surrounding taxation has a profound impact on attitudes towards welfare. When taxpayers erroneously perceive their tax burden to be lighter than it actually is, they become predisposed to champion expansive welfare programs without a holistic comprehension of their fiscal implications [70]. Consequently, individuals may support increased government spending without fully understanding the corresponding increase in taxation required to sustain such spending. This lack of understanding between taxation and welfare results in a cycle of fiscal irresponsibility, where the demand for increased benefits outstrips the willingness to bear the requisite tax burden [71].

Ability-to-pay serves as a principle in both tax law and economic theory, advocating for taxation to be commensurate with an individual’s financial capacity, which dictates that the tax burden should increase proportionally with one’s ability to pay, aligning tax liabilities with economic prowess. In essence, the design of a government’s tax system and the provision of public goods should be tailored to accommodate the diverse interests and financial capabilities of different societal strata and individuals, while also addressing their respective demands for social welfare [72]. The amount of tax should be allocated in a positive direction in relation to the taxpayer’s ability to pay. The design of the government’s tax system and the provision of public goods should consider the specific interests of different class groups and individuals, as well as their ability to bear the tax burden and their demands for social welfare [73]. As per Adam Smith’s proposal, every citizen should support the government to the best of their ability, in proportion to their income received under the protection of the state. Hence, taxes ought to be structured on the bedrock principle of "taxing according to ability". This mandates that individuals with similar tax capacities shoulder equivalent tax burdens, while those with disparate financial capacities should only bear taxes commensurate with their respective abilities. Consequently, those with greater financial means should contribute a larger share of their income, while those with lesser means should bear a comparatively lighter tax burden. Building upon these foundations, Edgeworth, drawing from Marginalist social welfare doctrine, posited that optimal taxation should strive to maximize societal welfare. This optimization hinges on a delicate balance, taking into account individual utility with diminishing marginal returns, juxtaposed against the proportional decrease in utility compared to income increments [74, 75]. Thus, the goal of taxation extends beyond mere revenue generation, aiming to enhance overall social well-being by judiciously allocating resources. Moreover, taxpayers’ assessment of tax policies is not solely driven by rational self-interest but also by their positioning relative to societal benchmarks in tax distribution and welfare benefits. In essence, taxpayers gauge the fairness of taxation by juxtaposing the magnitude of their tax burden against the benefits accrued from societal welfare programs. This comparative evaluation is influenced by a combination of societal consensus and individual self-appraisal, shaping perceptions of the adequacy and equity of the resources allocated to them. In other words, taxpayers assess the rationality of taxation by comparing the degree of tax burden to the welfare benefits received [76]. An individual’s perception of whether the social resources they receive are reasonable or not is based on a combination of social consensus and self-evaluation [77]. If individuals perceive their tax burden to exceed their personal capacity to bear, they are likely to demand a corresponding reduction in the scale of welfare provision by the government. This underscores the delicate balance policymakers must strike between taxation, welfare, and societal perceptions to foster a harmonious and equitable socio-economic landscape.

Based on the theoretical analysis presented above, individuals pay taxes as an exchange for accessing public goods provided by the government. However, if these goods fail to meet societal expectations, individuals may perceive the tax burden as unreasonable, potentially leading to negative attitudes towards welfare. Moreover, the concept of Fiscal Illusion suggests that individuals often miscalculate their actual tax burden, fostering disconnect between perceived tax burden and the value of public goods, which may also contribute to negative attitudes towards welfare. Additionally, the Ability-to-pay Principle underscores the importance of fair tax allocation, but perceptions of taxation exceeding one’s ability to pay can lead to dissatisfaction with welfare provision. Consequently, it is hypothesized that individuals’ perceived tax burden is negatively related to their attitudes towards welfare, as dissatisfaction with taxation and perceptions of unfairness may influence views on the efficacy and equity of welfare provision. This leads to the following hypotheses:

**H1:** Individuals’ tax burden is negatively related to their attitudes towards welfare.

### 2.3 Perceived fairness and welfare attitudes

Social justice is a fundamental concept that profoundly shapes societal attitudes toward government actions, particularly in the provision of public goods and social welfare [78]. It serves as a cornerstone of political legitimacy and affects the public’s perception of fairness in society. According to Frederickson [79], social justice is not just an aspirational goal but a critical aspect of governmental performance, akin to efficiency and effectiveness in public administration [80]. This perception of justice influences citizens’ expectations regarding achievement, wealth acquisition, and social mobility, consequently impacting their trust in government behaviors related to welfare provision and redistribution [81]. Social justice is intricately tied to distributive justice, which concerns the rational and legitimate allocation of social resources. This foundational principle of political justice shapes societal norms and expectations regarding fairness. The performance of the government is evaluated by citizens based on their perceived fairness in resource allocation and the extent to which they believe justice is upheld in society [82]. An individual’s perception of social fairness directly affects their recognition of political legitimacy [83, 84]. When citizens perceive the social system as fair, they are more likely to trust in governmental actions and have a positive attitude toward policies related to welfare provision. Conversely, the perception of injustices can result in a lack of trust and an increased demand for governmental intervention in the redistribution of resources and the provision of social welfare [85–87]. Social psychology offers further insights into the influence of perceptions of social fairness on attitudes towards welfare. Sociological institutionalism posits that institutions shape individuals’ preferences and self-identity, influencing their behaviours and attitudes towards social policies. Social comparisons play a crucial role in shaping individuals’ perceptions of their socioeconomic status relative to others, thereby impacting their support for welfare policies. Empirical studies by Schakel et al. confirm that perceived social fairness significantly influences redistributive preferences [88]. Individuals attribute responsibility for welfare based on their sense of social justice [89]. The relationship between social justice and welfare attitudes is also influenced by cultural and national values. In welfare states, shared cultural backgrounds and historical traditions shape societal norms regarding fairness and equality. For example, East Asian welfare states, which are influenced by Confucian values, place greater emphasis on familial responsibilities and have lower expectations of governmental involvement in social welfare compared to Western welfare models [40–42]. Research indicates that residents who perceive their society as fair and believe in the ability to achieve income through personal effort tend to favor moderate government involvement in welfare provision. They see market regulation as sufficient and prefer minimal governmental intervention in income distribution. Conversely, residents who perceive low levels of social justice and attribute economic success to factors like birth, luck, or corruption are more likely to support government intervention in income redistribution and social welfare provision.

Drawing upon theoretical frameworks from social justice, social culture, and social psychology, we derive the hypothesis that perceived fairness holds a negative relationship with welfare attitudes. Firstly, within the realm of social justice, justice is regarded as a fundamental value, shaping individuals’ perceptions of government legitimacy and their attitudes towards governmental actions, including welfare provision. When individuals perceive higher levels of fairness within their society, they may view government intervention in welfare provision as less necessary, leading to a decrease in support for extensive welfare policies [90]. Conversely, when fairness is perceived to be lacking, individuals may demand greater government involvement in welfare provision as a means to rectify perceived injustices, resulting in a more positive attitude towards welfare policies. Secondly, cultural factors play a significant role in shaping welfare attitudes. Different national cultures and values influence interpretations of fairness and justice, thereby impacting attitudes towards welfare provision. For instance, in societies with a strong emphasis on individual responsibility and self-reliance, individuals may view government welfare programs with skepticism, preferring minimal government intervention. In cultures where collective responsibility and social solidarity are prioritized, there may be greater support for expansive welfare policies as a means to ensure fairness and equality [91]. Therefore, perceptions of fairness within cultural contexts can influence the degree of support for government welfare interventions, with higher levels of perceived fairness associated with lower support for extensive welfare policies. Lastly, from a social psychology perspective, individuals’ perceptions of fairness shape their attitudes towards welfare provision through mechanisms such as social comparisons and institutional influences. When individuals perceive their society to be fair and meritocratic, they may attribute socioeconomic disparities to individual effort rather than systemic inequalities, leading to a reluctance to support extensive government intervention in welfare provision [92]. In contexts where perceived fairness is low, individuals may attribute socioeconomic disparities to systemic injustices, thereby advocating for greater government involvement in redistributive policies to address perceived inequalities. Thus, perceived fairness influences welfare attitudes by shaping individuals’ attributions of responsibility for welfare provision, with higher levels of perceived fairness associated with lower support for extensive government intervention in welfare provision.

In summary, across the realms of social justice, social culture, and social psychology, the hypothesis emerges that perceived fairness holds a negative relationship with welfare attitudes. Higher levels of perceived fairness are associated with lower support for extensive government intervention in welfare provision, while lower levels of perceived fairness are associated with greater support for government involvement in redistributive policies which leads to the research hypothesis 2:

**H2:** Perceived fairness have a negative relationship with welfare attitudes.

### 2.4 Tax burden and perceived fairness

The correlation between the tax burden and the perception of fairness reflects the intricate interplay between taxation policies, societal values, and individual perceptions, highlighting how people evaluate tax burdens in the context of broader notions of fairness and equity.

Taxation is a significant source of government revenue and a key measure of income redistribution, and it affects people’s subjective perceptions of the degree of social fairness [93]. It can promote social fairness by regulating income distribution through direct taxes. Simultaneously, promoting the fairness of the tax burden can also promote social fairness [94–96]. In the initial distribution link, economic and social activities often fail to provide equal opportunities due to individual differences and varying degrees of development between regions, sectors, or industries. This makes it impossible for the market to equalize the income gap between different groups of people, regions, and industries through resource allocation [97]. As a result, the sense of social fairness is negatively affected. The principle of fairness sometimes suggests direct recommendations regarding the relative weight of different income ranges [98]. A reasonable tax system is crucial for achieving relative equality in social welfare and public services, facilitating income regulation, and promoting the reasonable flow of wealth between developed and less developed regions, as well as high-income and low-income individuals, through a redistribution link and transfer payment system [99, 100].

The perception of tax fairness is intricately linked to societal norms and values, particularly those concerning social justice and equality. Societies that prioritize these principles tend to scrutinize tax burdens more rigorously, often favoring progressive tax systems to achieve a more equitable distribution of the burden among higher-income individuals. Within the tax relationship, the values of fairness and equality are paramount, as taxation inherently represents a form of authorization, reflecting the contractual relationship between citizens and the state. The rights and obligations of taxpayers and tax authorities are interrelated. Recognizing the power of taxation entails acknowledging taxpayers’ rights and fostering positive perceptions, emphasizing its role not just as a civic duty but also as a means for citizens to engage in societal development and access public services [101]. Transparency and accountability in tax policies are crucial for shaping and maintaining perceptions of fairness. A clear understanding of how tax revenues are allocated for public goods enhances public trust in the tax system. Conversely, opacity or mismanagement in tax policies can undermine this trust and raise doubts about tax fairness. Schmölders emphasizes tax fairness as a foundational aspect of tax morality [102]. Tax avoidance may be seen as unethical, as it seeks to evade societal contributions, yet it may also be viewed as a just response to societal injustice, particularly when the tax system itself exhibits unfairness. Nevertheless, the establishment of a fair, transparent, and efficient tax system is of paramount importance for the assurance of tax morality and societal equity [103].

Based on rational choice theory, individuals aim to maximize their own interests. Individuals assess the fairness of the tax burden based on factors such as the distribution of tax obligations across different income groups, the perceived benefits from public services and welfare programs, and the transparency and efficiency of tax collection and utilization processes. When these factors align with individuals’ expectations of fairness and equity, they are more likely to perceive the tax burden as justifiable and fair. Some scholars argue that a reasonable tax burden can enhance the sense of social fairness across four levels: Tax fairness can be divided into four aspects: rights fairness, which refers to the sense of fairness of taxpayers after comparing their own tax obligations with their rights; opportunity fairness, which refers to the sense of relative fairness of taxpayers after comparing their tax rights and obligations with those of other individuals in society; and process fairness, which refers to the sense of fairness of the process of collecting and using tax burdens; distributive fairness refers to the sense of fairness brought about by reasonable tax thresholds and tax rates set by law. A reasonable tax burden can improve the actual disposable income of low-income groups, enhance their sense of economic access and life satisfaction, narrow the wealth gap, reduce the sense of relative deprivation, and in the long run, improve individuals’ subjective expectations of social class mobility and enhance their sense of social justice [104].

The correlation between the tax burden and the perception of fairness is multifaceted. Taxation policies not only shape income distribution but also impact societal perceptions of fairness and equity. Societal norms and values, along with transparency and accountability in tax policies, further shape perceptions of fairness. Rational choice theory suggests that individuals evaluate the fairness of tax burdens based on various factors, including distribution, benefits from public services, and efficiency in tax processes. A reasonable tax burden can enhance social fairness across different dimensions, ultimately improving individuals’ subjective expectations of social justice. Therefore, we propose the following research hypothesis:

**H3:** A positive correlation exists between the tax burden and the perception of fairness.

### 2.5 Government efficacy

Government efficacy, a pivotal concept in political science and public administration, denotes the perceived effectiveness and efficiency of governmental institutions in enacting policies, managing public resources, and delivering essential services to the populace [105]. High government efficacy implies a government that is viewed as competent, trustworthy, and capable of managing public funds effectively. Conversely, low government efficacy indicates perceptions of governmental incompetence, corruption, and mismanagement [106].

The perceived efficacy of government plays a crucial moderating role in shaping public attitudes towards various policy issues, including welfare policies, particularly in the context of tax burdens [107]. When the government is perceived as highly efficacious, citizens are more likely to believe that their tax contributions are being utilized judiciously and effectively. In such contexts, even a high tax burden might not significantly diminish public support for welfare policies. Citizens may be inclined to support extensive welfare policies because they trust the government to deploy funds efficiently to improve public welfare. This trust can buffer the negative impact of a high tax burden on welfare attitudes, maintaining or even enhancing public support for welfare policies. In societies where government efficacy is perceived to be high, the public is more likely to see the value in their tax contributions, fostering a positive feedback loop where effective government performance legitimizes higher taxation and robust welfare programs. In contrast, when the government is perceived as ineffective or corrupt, a high tax burden can exacerbate negative attitudes towards welfare policies [108]. Taxpayers may feel that their money is being wasted or misused, leading to increased resistance to welfare spending [109]. Low government efficacy amplifies the negative impact of a high tax burden on welfare attitudes, decreasing public support for social programs. In such contexts, citizens are likely to be skeptical about the government’s ability to manage public funds effectively, resulting in opposition to both higher taxes and welfare policies [110].

Government efficacy plays a moderating role in shaping public attitudes towards welfare policies and tax burdens. High government efficacy can buffer the negative impact of high taxes on welfare attitudes, maintaining or even enhancing public support for social programs. Low government efficacy amplifies the negative impact of high taxes on welfare attitudes, leading to decreased public support for welfare policies. This leads to the following hypotheses:

**H4:** Government efficacy negatively moderates the impact of tax burden on welfare attitudes.

## 3 Methodology and data

### 3.1 Model

This study’s empirical analysis aims to prove the impact of tax burden on welfare attitudes. We create a benchmark regression model, is as follows:

$$attitude_{i}=\alpha_{0}+\alpha_{1}taxburden_{i}+\alpha_{2}control_{i}+\mu_{i}+\varepsilon_{i}$$ (1)

In Eq (1), the explanatory variable *attitude* is the welfare attitude of the individuals; the explanatory variable *taxburden* indicates the perceived tax burden of the individuals; *control* is a series of control variables, which mainly include, gender, age, marital status, education attainment, household size, number of children, social class, and employment status; *μ* indicates a fixed effect of region; *ε* is the random error term; *i* represents the individuals in the sample. The sign and significance of *α*_*1*_ and *α*_*2*_ require our attention.

The Ordered Probit (OProbit) statistical method is considered an a priori optimal selection for the current investigation, stemming from several rationales [111, 112]. First, welfare attitudes are often measured on an ordinal scale, with respondents selecting from categories like "strongly agree," "agree," "neutral," "disagree," and "strongly disagree." These categories represent a natural ordering, as "strongly agree" is considered a more positive attitude than "agree," and so forth. The OProbit method is designed to model such ordinal responses, effectively capturing the inherent structure of the data. Moreover, in empirical studies, especially those based on survey data, the assumption of normality in the error term may not hold. The OProbit method does not require adherence to the normality assumption and is robust to deviations from it. This flexibility is crucial, as welfare attitudes can exhibit diverse patterns and may not strictly adhere to a normal distribution. Furthermore, OProbit provides coefficients that are interpretable in that they reflect the impact of independent variables on the likelihood of observing different levels of the ordinal dependent variable. For instance, a positive coefficient for tax burden would indicate an increase in the likelihood of more positive welfare attitudes, while a negative coefficient would suggest the opposite. This clarity in interpretation enhances the practical relevance of the results. In addition, the OProbit model can accommodate both categorical and continuous predictors, rendering it suitable for analyzing the effects of tax burden and control variables on welfare attitudes. This flexibility allows researchers to incorporate a wide range of individual characteristics into the analysis, thereby capturing the multifaceted determinants of welfare attitudes. Finally, OProbit is robust to potential sample selection bias, which may arise when individuals with certain characteristics are more likely to participate in surveys or studies. By accounting for the ordinal nature of the dependent variable and controlling for relevant covariates, OProbit helps mitigate the risk of biased estimates and enhances the validity of the findings. The selection of OProbit as the statistical method in this study reflects a considered approach to the ordinal nature of welfare attitudes, the potential challenges in modelling them, and the need for robust and interpretable results. By leveraging the strengths of OProbit, the study aims to provide rigorous insights into the relationship between tax burden and welfare attitudes, contributing to a deeper understanding of this complex phenomenon.

### 3.2 Data source

Welfare attitudes are commonly assessed using standardized, closed-ended questionnaires and random sampling statistical methods [113]. Currently, there are several representative large-scale social surveys available, such as the International Social Survey Program (ISSP), the World Values Survey (WVS), the Eurobarometer, and the European Social Survey (ESS), which include questions on attitudes towards social welfare. It is notable that these measures are subjective indicators.

The ISSP is a cross-national collaborative program that conducts annual surveys on various topics related to the social sciences. The ISSP 2019 Social Inequality V is the latest survey that includes attitudes towards social welfare. Researchers can use these survey databases to construct a multi-level index system of social welfare attitudes and expand the connotation and extension of welfare attitudes.

In this study, we analyze data from the ISSP 2019 in 11 welfare countries and regions [114]. In sample selection, we adopted the categorization method of Esping-Andersen (1990) and subsequent scholars for typical welfare state. Specifically, after proposing missing values to select valid samples, we included the United States (n = 1389), the United Kingdom (n = 1280), and New Zealand (n = 915) as liberal welfare states; France (n = 1155), Germany (n = 595), and Italy (n = 1060) as conservative welfare states; Sweden (n = 1050), Norway (n = 988), and Denmark (n = 721) as social-democratic welfare states, and Japan (n = 793), and China-Taiwan (n = 1355) as East Asian welfare states (societies), for a total sample size of 11301.

### 3.3 Variables

#### 3.3.1 Explained variable

*Welfare attitudes*. The welfare attitude measure assesses the degree to which individuals depend on the government for welfare provision and social redistribution. We use question Q4b of the questionnaire: the questionnaire item asks respondents to rate their level of agreement with the statement "It is the responsibility of the government to reduce the differences in income between people with high incomes and those with low incomes." using a scale of 1 to 5, with 1 being "strongly agree" and 5 being "strongly disagree". A higher rating indicates a lower level of dependence on government redistribution. To facilitate the interpretation of the empirical results, this study assigns a reverse value to the question, ranging from 1 to 5, with 1 representing "strongly disagree" and 5 representing "strongly agree".

#### 3.3.2 Explanatory variable

*Tax burden*. Tax burden is usually understood as an individual’s subjective assessment of the tax pressure that he or she has to endure. In this study, the value of tax burden was selected from question Q8b in the survey questionnaire: "Generally, how would you describe taxes in [COUNTRY] today for those with high incomes? Taxes are…" Respondents had the following valid options: "1 = much too high," "2 = too high," "3 = about right," "4 = too low," and "5 = much too low." According to the ability-to-pay principle, the higher the taxpayer’s ability to pay, the higher the amount of tax they should bear. In addition, the tax burden as a proportion of total income should increase accordingly. Thus, the increase in perceived tax burden among high-income earners suggests that the distribution of tax burden and the overall tax mechanism are appropriate and rational. In the empirical analysis, this study assigns a reverse value to the options, from 1 to 5, with a higher value indicating greater rationality of the tax burden.

#### 3.3.3 Control variables

The control variables affecting residents’ welfare attitudes are individual characteristic factors, family characteristic factors and social characteristic factors. In this study, individual characteristics include gender, age, marital status and education attainment; family characteristics include household size and number of children; social characteristics include social class and employment status.

1. **Gender:** a dummy variable, assigned a value of 1 for males and 0 for females.
2. **Age:** a continuous variable, the age of the respondent, taking the value of 18–99.
3. **Marital status:** a dummy variable, with a value of 1 assigned to the status of being married and 0 assigned to other marital statuses.
4. **Educational attainment:** a ordinal variable, ranging from 0 to 6 for "no education" to "master’s or doctoral degree", with higher values indicating higher levels of education.
5. **Household size:** a continuous variable, filled in by the respondents according to the actual household situation, with a minimum value of 1.
6. **Number of children:** a continuous variable, refers to the total number of children of the respondent.
7. **Social class:** a ordinal variable, derived from the questionnaire Q22 "which social class you would say you belong to?", the options were "1 = lower class", "2 = working class", "3 = lower middle class", "4 = middle class", "5 = upper middle class", "6 = upper class", the higher the value, the higher the self-assessed social class.
8. **Employment status:** a dummy variable, the data were generalized from the respondents’ answers on employment status in the questionnaire, in which "I am now in a paid job" is assigned the value of 1, and the rest of the work status is assigned the value of 0.

#### 3.3.4 Mediator variable

*Perceived fairness*. We adopt question Q16 in the questionnaire "How fair or unfair do you think the income distribution is in [COUNTRY]?" to reflect the individual residents’ perception of social fairness. The respondents’ valid options include "1 = very fair", "2 = fair", "3 = unfair", "4 = very unfair". Similarly, for the purpose of empirical analysis and interpretation, we assign values to the options in reverse order, with larger values representing higher levels of social equity as perceived by the respondents.

#### 3.3.5 Moderator variable

*Government efficacy*. We adopt question Q6 in the questionnaire "Most politicians in [COUNTRY] do not care about reducing the differences in income between people with high incomes and people with low incomes." to reflect the respondents’ evaluation of government efficacy. The respondents’ valid options include "1 = strongly agree", "2 = agree", "3 = neither agree nor disagree", "4 = disagree", "5 = strongly disagree". The higher the score, the higher the public’s perception of government efficacy.

The descriptive statistics for each variable are provided in **Table 1**, offering an overview of their distribution and key characteristics for further analysis.

**Table 1:** Descriptive statistics of variables.

Variable	Sample Size	Mean	Standard Deviation	Minimum Value	Maximum Value	Interquartile Range
Welfare attitude	11301	3.657	1.144	1	5	1
Tax burden	11301	2.702	1.111	1	5	1
Gender (Male = 1)	11301	0.502	0.5	0	1	1
Age	11301	52.064	17.069	18	98	27
Marital status (Married = 1)	11301	0.547	0.498	0	1	1
Educational attainment	11301	3.889	1.525	0	6	2
Household size	11301	2.742	1.473	1	15	2
Number of children	11301	0.537	0.955	0	8	1
Social class	11301	3.381	1.096	1	6	2
Employment status (Employed = 1)	11301	0.601	0.49	0	1	1
Perceived fairness	11301	2.135	0.694	1	4	1
Government efficacy	11301	2.137	0.993	1	5	2

## 4 Results

### 4.1 Benchmark regression

For the basic empirical analysis, the explanatory variable *welfare attitude* is assigned a fixed-order variable from 1 to 5, indicating low to high welfare attitude. This makes it suitable for regression estimation using Ordered Probit model. Using the Eq (1) constructed in the previous section, we performed a stepwise regression with Stata 17.0 to obtain benchmark regression results for tax burden and welfare attitude. We focus on the direction and significance of the regression coefficient signs in the benchmark regression results, as the specific values of the coefficients in the Oprobit model hold no practical significance.

**Table 2,** column (1), displays the univariate regression results. The regression coefficient of tax burden on welfare attitude preference is -0.314, which is significant at the 1% level. This indicates a negative correlation between the reasonableness of the tax burden and welfare attitude. When residents perceive the tax burden as unreasonable, they rely more heavily on government redistribution. Residents’ reliance on government redistribution is inversely proportional to their perception of the tax burden’s reasonableness. In columns (2) to (4), we gradually add the control variables of individual characteristic factors, family characteristic factors, and social characteristic factors to the regression model, and the results show that the regression coefficients are -0.322, -0.322, and -0.309, respectively, which are significant at the 1% level, and are consistent with the direction of the sign and the significance of the results in Column (1), which indicate that the relationship between the reasonableness of tax burden and the welfare attitudes presents a significant negative correlation.

**Table 2:** Benchmark regression results.

	(1)	(2)	(3)	(4)
Variable	Welfare attitude	Welfare attitude	Welfare attitude	Welfare attitude
Tax burden	-0.314***	-0.322***	-0.322***	-0.309***
	(-27.35)	(-27.96)	(-27.86)	(-26.72)
Gender		-0.165***	-0.167***	-0.164***
		(-8.15)	(-8.22)	(-8.05)
Age		-0.00433***	-0.00448***	-0.00469***
		(-6.70)	(-6.21)	(-6.00)
Marital status		-0.0772***	-0.0781***	-0.0495**
		(-3.61)	(-3.39)	(-2.14)
Education level		-0.0423***	-0.0414***	-0.00607
		(-5.99)	(-5.85)	(-0.79)
Family size			0.0187*	0.0235**
			(1.65)	(2.06)
Number of children			-0.0395**	-0.0423***
			(-2.52)	(-2.68)
Social class				-0.118***
				(-10.78)
Employment status				-0.0710***
				(-2.98)
Regional fixed effects	Yes	Yes	Yes	Yes
/				
cut1	-2.405***	-2.927***	-2.883***	-3.115***
	(-55.62)	(-46.14)	(-36.47)	(-36.97)
cut2	-1.655***	-2.167***	-2.123***	-2.349***
	(-39.48)	(-35.03)	(-27.24)	(-28.26)
cut3	-1.084***	-1.590***	-1.545***	-1.768***
	(-27.04)	(-26.25)	(-20.10)	(-21.54)
cut4	0.0558	-0.441***	-0.396***	-0.611***
	(1.45)	(-7.46)	(-5.23)	(-7.56)
N	11301	11301	11301	11301
Pseudo R2	0.045	0.051	0.051	0.055

Note: *, **, and *** indicate statistical significance at the 10%, 5%, and 1% levels, respectively.

Gender, age, and marital status significantly affect welfare attitudes, while education does not have a significant effect among the individual characteristic factors of the control variables. Possible reasons for this difference in reliance on government protection between men and women may be due to women’s tendency to consider family risks more carefully, while men may focus more on family development. Additionally, as individuals age and attain higher levels of education, their economic autonomy increases, leading to a decreased demand for government welfare support. The impact of education level becomes non-significant after controlling for family and social characteristics. This may be because factors such as family size, number of children, social class, and employment status have a greater impact on individuals’ economic and overall development, which dilutes the effect of changes in education on welfare attitudes and leads to insignificant results.

Regarding the family characteristics factors of the control variables, it was found that family size had a negative and significant effect and the number of children showed a negative and significant effect. Larger populations have relatively large intra-household burdens and an increased likelihood of uncertain risks, making members of larger households more likely to place welfare responsibilities on the state and government. The impact of the number of children on welfare attitudes is significant due to the low fertility rate typical of welfare states. The number of children tends to follow a U-shaped distribution according to income class, with both low-income earners and high-income earners showing a stronger desire to have children. Low-income earners can rely on their families for support, while high-income earners have greater financial resources, making them less likely to attribute welfare responsibility to the government.

Regarding the social characteristics of the control variables, both social class and employment status have a negative effect on welfare attitudes. This may be due to the fact that as social class and employment status increase, individuals’ economic disposability and resource mobilization ability also increase, leading to a greater ability to resist risk and a decreased dependence on government welfare redistribution.

The empirical results indicate that a reasonable tax burden can have a negative impact on residents’ attitudes towards welfare. In other words, residents who perceive the current tax burden as reasonable are less likely to attribute the responsibility of redistributing welfare to the government, thus supporting **H1** of this study.

### 4.2 Robustness test

#### 4.2.1 Regression using OLS and OLogit models

The benchmark regression employs the Oprobit model, which shows that a moderate tax burden can discourage residents from relying on government welfare redistribution. To test the reliability of this conclusion and determine whether it is affected by model selection factors, relevant studies have confirmed that regression results using OLS and OLogit should not significantly differ from those derived from the original model in terms of the significance and direction of the regression coefficients. This study’s explanatory variables are discrete and ordered variables. Therefore, this study employs OLS and OLogit models for robustness testing. Table 3 below reports the regression results, with columns (1) and (2) displaying the OLS regression results and columns (3) and (4) displaying the OLogit regression results. Compared to the benchmark regression results obtained from the OProbit model in the previous section, the regression coefficients of the variables remain unchanged in terms of both direction and significance level. This suggests that the study’s findings are robust.

**Table 3:** Robustness test results using OLS and OLogit models.

	(1)	(2)	(3)	(4)
Variable	OLS	OLS	OLogit	OLogit
Tax burden	-0.317***	-0.307***	-0.552***	-0.543***
	(-29.33)	(-28.73)	(-26.81)	(-26.20)
Gender		-0.178***		-0.281***
		(-8.91)		(-7.98)
Age		-0.00465***		-0.00790***
		(-6.13)		(-5.85)
Marital status		-0.0513**		-0.0880**
		(-2.25)		(-2.21)
Education level		-0.00587		-0.00618
		(-0.77)		(-0.46)
Family size		0.0242**		0.0406**
		(2.17)		(2.05)
Number of children		-0.0389**		-0.0769***
		(-2.50)		(-2.83)
Social class		-0.113***		-0.204***
		(-10.64)		(-10.69)
Employment status		-0.0705***		-0.119***
		(-3.05)		(-2.90)
Regional fixed effects	Yes	Yes	Yes	Yes
_cons	4.356***	5.012***		
	(112.73)	(63.51)		
/				
cut1			-4.237***	-5.434***
			(-53.84)	(-36.70)
cut2			-2.834***	-4.009***
			(-37.48)	(-27.44)
cut3			-1.868***	-3.024***
			(-26.12)	(-21.04)
cut4			0.0176	-1.105***
			(0.26)	(-7.85)
N	11301	11301	11301	11301
R^2^ / Pseudo R^2^	0.126	0.151	0.126	0.151

Note: *, **, and *** indicate statistical significance at the 10%, 5%, and 1% levels, respectively.

#### 4.2.2 Alternative measures of welfare attitudes

In response to the study’s definition of welfare attitudes as residents’ preference for the government’s role in the redistribution process, we selected question Q5 of the questionnaire: "Who do you think should have the greatest responsibility for reducing differences in income between people with high incomes and people with low incomes?", and the options available to the respondents include "1 = Private companies," "2 = Government," "3 = Trade unions," "4 = High-income individuals themselves," "5 = Low-income individuals themselves," "6 = Income differences do not need to be reduced," "8 = Unable to choose". To analyze the respondents’ preference for the role of the government, we transformed the question options into dichotomous variables. The option "government" was assigned a value of 1, while the other options were assigned a value of 0. We then assigned the same values to the remaining variables based on their dichotomous nature and performed regression estimation on the baseline model using both Probit and Logit methods. The table below presents the regression results. In **Table 4,** columns (1) and (2) display the Probit regression results for the core explanatory variables and the control variables, respectively. Columns (3) and (4) show the Logit regression results for the core explanatory variables and the control variables, respectively. The regression coefficients for the explanatory variable tax burden are consistent in sign direction and significance with the benchmark regression, indicating that the previous study’s findings are robust.

**Table 4:** Robustness test results with alternative explained variables.

	(1)	(2)	(3)	(4)
Variable	OLS	OLS	OLogit	OLogit
Tax burden	-0.317***	-0.307***	-0.552***	-0.543***
	(-29.33)	(-28.73)	(-26.81)	(-26.20)
Gender		-0.178***		-0.281***
		(-8.91)		(-7.98)
Age		-0.00465***		-0.00790***
		(-6.13)		(-5.85)
Marital status		-0.0513**		-0.0880**
		(-2.25)		(-2.21)
Education level		-0.00587		-0.00618
		(-0.77)		(-0.46)
Family size		0.0242**		0.0406**
		(2.17)		(2.05)
Number of children		-0.0389**		-0.0769***
		(-2.50)		(-2.83)
Social class		-0.113***		-0.204***
		(-10.64)		(-10.69)
Employment status		-0.0705***		-0.119***
		(-3.05)		(-2.90)
Regional fixed effects	Yes	Yes	Yes	Yes
_cons	4.356***	5.012***		
	(112.73)	(63.51)		
/				
cut1			-4.237***	-5.434***
			(-53.84)	(-36.70)
cut2			-2.834***	-4.009***
			(-37.48)	(-27.44)
cut3			-1.868***	-3.024***
			(-26.12)	(-21.04)
cut4			0.0176	-1.105***
			(0.26)	(-7.85)
N	11301	11301	11301	11301
R^2^ / Pseudo R^2^	0.126	0.151	0.126	0.151

Note: *, **, and *** indicate statistical significance at the 10%, 5%, and 1% levels, respectively.

#### 4.2.3 Primary explanatory variable validation with 2SLS

To mitigate potential endogeneity issues, we employs instrumental variable (IV) and two-stage least squares (2SLS) regression methods. Instrumental variables are exogenous variables that are correlated with the endogenous explanatory variable of interest but not with the error term [115, 116]. As a result, they are suitable for identifying the causal effect of the independent variable on the outcome of interest. In this study, the tax measurement of high-income individuals are utilized as instrumental variables for tax burden. These measurements directly affect tax burden but are assumed to be unrelated to welfare attitudes, thus meeting the exogeneity criterion. We adopted Question Q8a "Do you think people with high incomes should pay a larger share of their income in taxes than those with low incomes, the same share, or a smaller share?" The available options are: "1 = Much larger share," "2 = Larger," "3 = The same share," "4 = Smaller," and "5 = Much smaller share." It is important to note that since the question is a judgment of the respondents on the contingent outcome, the current actual situation is opposite to the tendency expressed by the answered options, for example, in option 5, the respondents chose "a smaller proportion", which means that they think that the current tax on the high-income group is too high. According to the definition of reasonable tax burden in the previous section, the higher income class should bear a greater share of the tax burden. Therefore, the higher the value assigned to this question, the more reasonable the tax burden. The respondents’ answers to this question are significantly related to the tax burden but do not directly influence attitudes toward welfare. Such variables can therefore be served as an instrumental variable (tax burden IV) for the explanatory variable tax burden.

Subsequently, the two-stage least squares (2SLS) method is employed to examine the robustness of the regression for the primary dependent variable. The 2SLS approach, comprises two stages. In the initial stage, the instrumental variables are regressed on the potentially endogenous explanatory variable to obtain predicted values, which are subsequently employed in lieu of the original endogenous variable in the second-stage regression model. This two-stage process serves to mitigate endogeneity by isolating the variation in the explanatory variable that is unrelated to the error term. The 2SLS results in **Table 5** confirm the robustness of the original conclusions, as the regression coefficients of the explanatory variables have the same direction and significance as in the benchmark regression.

**Table 5:** 2SLS results with instrumental variable.

	(1)	(2)
Variable	Tax burden	Welfare attitude
Tax burden IV	0.3172***	
	(0.0174)	
Tax burden		-0.9750***
		(0.0623)
Control variable	Yes	Yes
Regional fixed effects	Yes	Yes
N	11301	11301
R^2^ / Pseudo R^2^	-	-0.1958

Note: *, **, and *** indicate statistical significance at the 10%, 5%, and 1% levels, respectively.

## 5 Heterogeneous effects analysis

### 5.1 Examining the effects of different welfare regimes

Scholars have examined the institutional logic that underlies welfare attitudes, revealing differences in citizens’ attitudes towards welfare across different welfare regimes [6, 117–119]. Welfare institutions are significant external macro-factors that influence citizens’ attitudes towards welfare. Existing studies and discussions on the impact of different welfare regimes on welfare attitudes are often based on the typological division of the three worlds of welfare capitalism proposed by Esping-Andersen. This classification system categorizes welfare state regimes into the liberal welfare state, the conservative welfare state, and the social-democratic welfare state based on the degree of decommodification of the welfare state, where individuals do not have to rely on the sale of their labor force to sustain their livelihoods. Different welfare regimes create distinct structures within specific social groups. Within these three welfare regimes, the degree of decommodification decreases from liberal to conservative to social democratic [120]. According to Linos and West, citizens under liberal welfare regimes tend to have an individualistic attitude towards welfare, while those under social democratic welfare regimes believe that the government has a greater responsibility for welfare, citizens under conservative welfare regimes hold attitudes towards welfare that fall somewhere in between [121]. The level of support for government provision of welfare increases sequentially from liberalism, to conservatism, to social democracy. However, some scholars disagree and argue that the relationship between social welfare institutions and welfare attitudes is not entirely inverse. They suggest that citizens’ welfare attitudes do not vary significantly across different welfare institutions, particularly in areas related to minimum subsistence or basic health security, where differences in attitudes are minimal.

Some researchers have subdivided welfare regimes into empirical indicators, such as social welfare expenditure, Gini coefficient, female labor market participation rate, degree of de-commoditization, and employers’ social security contribution rate. They found that the degree of citizens’ support for the government’s income redistribution policy is significantly affected by welfare regimes. The support ranges from conservative, social democratic, and liberal welfare regimes in the order of high to low, respectively [34]. Meanwhile, as a complement and development of the Esping-Anderson typology of welfare regimes, the concept of East Asian welfare regimes has been proposed in the academic community. Despite being controversial, East Asian welfare regimes are characterized by productivism, familism, and a strong influence of Confucianism, making them a unique unit of research and analysis that distinguishes them from Western welfare regimes. Studies have shown that East Asian welfare regimes tend to have low levels of social welfare expenditure, but high levels of social investment expenditure and welfare stratification in terms of people’s welfare attitudes [40].

We formed clusters of the four welfare regimes and put them into the model for regression. **Table 6** presents the regression results, which indicate that tax burden has a negative and significant effect on welfare attitudes at the 1% level. These findings support the conclusions drawn in the previous section and suggest that welfare regimes are not the only determinants of welfare attitudes. It is important to note that there are individual differences in the control variables for different welfare regimes, which can affect expectations of government responsibility for welfare.

**Table 6:** Heterogeneity analysis results under different welfare regimes.

	(1)	(2)	(3)	(4)
Variable	Liberalism	Conservatism	Social democracy	East Asian model
Tax burden	-0.306***	-0.0614***	-0.479***	-0.266***
	(-15.87)	(-3.61)	(-17.70)	(-9.65)
Gender	-0.0719**	-0.153***	-0.281***	-0.129***
	(-2.00)	(-3.71)	(-6.73)	(-2.74)
Age	-0.00843***	-0.00240	-0.00659***	0.000705
	(-6.57)	(-1.44)	(-3.83)	(0.38)
Marital status	-0.0853**	-0.0179	-0.0520	-0.0520
	(-2.11)	(-0.37)	(-1.05)	(-0.94)
Educational attainment	-0.00386	-0.0643***	-0.00414	-0.00567
	(-0.29)	(-4.20)	(-0.27)	(-0.28)
Household size	0.0555**	0.0146	-0.0000193	0.0267*
	(2.47)	(0.55)	(-0.00)	(1.65)
Number of children	-0.0603**	-0.0621*	-0.0209	-0.0583*
	(-2.04)	(-1.75)	(-0.56)	(-1.82)
Social class	-0.141***	-0.162***	-0.144***	-0.0410*
	(-7.55)	(-7.00)	(-6.15)	(-1.71)
Employment status	-0.141***	-0.0753	-0.0206	-0.0422
	(-3.39)	(-1.54)	(-0.41)	(-0.79)
/				
cut1	-3.245***	-3.262***	-4.196***	-2.594***
	(-24.22)	(-19.20)	(-23.27)	(-14.25)
cut2	-2.585***	-2.555***	-3.403***	-1.617***
	(-19.87)	(-15.65)	(-19.59)	(-8.95)
cut3	-1.973***	-1.958***	-2.747***	-1.198***
	(-15.38)	(-12.16)	(-16.05)	(-6.68)
cut4	-0.923***	-0.801***	-1.470***	-0.0290
	(-7.33)	(-5.04)	(-8.84)	(-0.16)
N	3584	2810	2759	2148
Pseudo R^2^	0.045	0.022	0.084	0.023

Note: *, **, and *** indicate statistical significance at the 10%, 5%, and 1% levels, respectively.

At the same time, we analyze the results of the regressions in specific countries. **Table 7** shows that our conclusions still hold and that the tax burden still has a negative impact on welfare attitudes in most of the countries in the sample. Among them, the results for Italy do not show a significant correlation. This may be related to Italy’s unique economic structure and socio-cultural context, which might result from a complex interplay of several factors, such as a complex tax system, a generalized and equalized welfare system, a large informal economy, low trust in government due to cultural and historical factors, unbalanced regional economic development, and social inequality. These factors make it difficult for people to intuitively link tax burdens to benefits, thus weakening the significant correlation.

**Table 7:** Regression results in different countries.

	(1)	(2)	(3)	(4)	(5)	(6)	(7)	(8)	(9)	(10)	(11)
Variable	United States	Great Britain	New Zealand	Germany	France	Italy	Sweden	Norway	Denmark	Japan	China-Taiwan
Tax burden	-0.446***	-0.253***	-0.227***	-0.198***	-0.356***	-0.0192	-0.451***	-0.492***	-0.505***	-0.283***	-0.238***
	(-13.99)	(-7.38)	(-5.29)	(-4.08)	(-9.33)	(-0.71)	(-10.78)	(-10.26)	(-9.39)	(-7.02)	(-6.18)
Gender	-0.0729	-0.0887	-0.0724	-0.221**	-0.0945	-0.153**	-0.255***	-0.290***	-0.293***	-0.116	-0.133**
	(-1.25)	(-1.46)	(-1.01)	(-2.46)	(-1.47)	(-2.20)	(-3.66)	(-4.17)	(-3.65)	(-1.48)	(-2.21)
Age	-0.0164***	-0.00624***	0.000192	-0.000726	-0.00392	-0.0000906	-0.0144***	-0.00352	-0.00182	-0.00114	0.00240
	(-8.05)	(-2.70)	(0.07)	(-0.19)	(-1.21)	(-0.03)	(-4.84)	(-1.21)	(-0.55)	(-0.38)	(0.95)
Marital status	-0.188***	-0.0850	-0.107	0.0441	-0.106	0.0493	-0.0156	-0.0101	-0.175*	0.0222	-0.106
	(-2.81)	(-1.17)	(-1.36)	(0.40)	(-1.37)	(0.59)	(-0.20)	(-0.12)	(-1.75)	(0.24)	(-1.49)
Educational attainment	0.0501*	0.0174	0.0428*	-0.0807*	-0.0197	-0.0649**	-0.0464**	0.0356	0.0192	-0.0627	0.0250
	(1.95)	(0.73)	(1.84)	(-1.78)	(-0.89)	(-2.30)	(-2.09)	(1.36)	(0.45)	(-1.55)	(0.97)
Household size	0.0852**	0.0535	0.107***	-0.0974	0.0470	-0.0171	0.00703	0.0168	0.0117	0.0150	0.0314
	(2.05)	(1.13)	(2.90)	(-1.40)	(0.96)	(-0.41)	(0.12)	(0.31)	(0.19)	(0.36)	(1.64)
Number of children	-0.141***	-0.00462	-0.0717	0.0457	-0.0406	-0.0424	-0.0825	-0.00960	0.00939	-0.0884	-0.0312
	(-2.80)	(-0.08)	(-1.34)	(0.56)	(-0.66)	(-0.71)	(-1.27)	(-0.15)	(0.13)	(-1.52)	(-0.76)
Social class	-0.0862***	-0.162***	-0.196***	-0.209***	-0.183***	-0.0187	-0.118***	-0.159***	-0.177***	-0.0681	-0.0277
	(-2.99)	(-4.92)	(-5.10)	(-3.94)	(-4.82)	(-0.49)	(-3.04)	(-4.20)	(-3.69)	(-1.58)	(-0.94)
Employment status	-0.124*	-0.155**	-0.195**	0.0459	-0.0747	-0.113	-0.0194	-0.0111	-0.0591	0.0366	-0.0937
	(-1.92)	(-2.13)	(-2.30)	(0.40)	(-0.89)	(-1.44)	(-0.23)	(-0.14)	(-0.61)	(0.39)	(-1.39)
/											
cut1	-3.339***	-3.343***	-2.604***	-3.896***	-3.818***	-3.157***	-4.557***	-4.091***	-3.998***	-2.697***	-2.661***
	(-15.44)	(-14.20)	(-8.59)	(-9.26)	(-13.70)	(-10.56)	(-15.24)	(-13.52)	(-10.96)	(-8.19)	(-11.19)
cut2	-2.696***	-2.643***	-1.803***	-3.052***	-3.108***	-2.317***	-3.910***	-3.197***	-3.091***	-2.204***	-1.246***
	(-12.76)	(-11.58)	(-6.14)	(-7.56)	(-11.53)	(-8.56)	(-13.38)	(-11.15)	(-8.78)	(-6.75)	(-5.46)
cut3	-2.024***	-1.978***	-1.222***	-2.587***	-2.402***	-1.618***	-3.198***	-2.528***	-2.488***	-1.451***	-1.012***
	(-9.78)	(-8.77)	(-4.19)	(-6.56)	(-9.04)	(-6.02)	(-11.19)	(-8.88)	(-7.19)	(-4.49)	(-4.45)
cut4	-1.097***	-0.768***	-0.118	-1.411***	-1.345***	-0.213	-1.897***	-1.079***	-1.459***	-0.610*	0.360
	(-5.40)	(-3.46)	(-0.41)	(-3.67)	(-5.12)	(-0.80)	(-6.91)	(-3.89)	(-4.30)	(-1.91)	(1.59)
N	1389	1280	915	595	1155	1060	1050	988	721	793	1355
Pseudo R2	0.092	0.032	0.033	0.043	0.055	0.011	0.093	0.076	0.082	0.037	0.016

Note: *, **, and *** indicate statistical significance at the 10%, 5%, and 1% levels, respectively.

### 5.2 Examining the effects of different social classes

An individual’s self-interest is often influenced by their socio-economic class. Individuals from varying socio-economic backgrounds possess distinct social resources and opportunities for development, and hold differing definitions and standards of self-interest. These factors contribute to varying attitudes towards state welfare provision [23, 28, 34, 122–124]. Social class may be negatively correlated with welfare preferences. Groups with higher socioeconomic status may be less concerned with and supportive of government welfare policies. They may wish to limit government intervention in the redistributive sphere and rely more on the market to play a regulatory role. This is while perceiving the necessity of the existence of income and class inequality [30, 125]. In contrast, individuals from lower social strata can gain a lot from welfare policy, which leads to a positive attitude towards the institutional arrangements of the state and welfare policy. The attitudes of individuals towards social welfare are influenced by their relative position in the system. Welfare contributors, who pay high taxes or social security fees but do not directly benefit from social welfare protection, tend to have negative attitudes towards welfare policies. On the other hand, welfare recipients, who have lost their jobs or are living in poverty, are usually more supportive of government intervention in social welfare provision. Welfare practitioners and employees, who work in public social security institutions and rely on welfare for their livelihoods, also have a stake in the system. It is important to consider these different perspectives when evaluating social welfare policies. According to Andreβ and Heien [19], individuals who rely on welfare for their income and have knowledge of the internal processes of welfare institutions are more likely to value fairness and justice, leading to a positive attitude towards welfare policy.

High-income individuals have a greater economic ability and higher risk tolerance. They also experience a lower tax burden compared to low-income individuals, despite paying the same amount of taxes. As wealth accumulates, the marginal utility of social welfare gradually decreases. Conversely, low-income individuals have weaker tax-paying abilities and experience a stronger tax burden. The marginal utility of social welfare is more apparent for them. Although the low-income class and the high-income class may pay the same tax, the tax burden and perception of welfare satisfaction differ due to the proportion of income accounted for by the tax and the marginal utility of the benefits received. This can lead to a sense of relative deprivation among different income classes.

We formed clusters of the different social class and put them into the model for regression. **Table 8** shows the regression results, indicating that the tax burden has a negative and statistically significant impact on the welfare attitudes of the working class, lower middle class, middle class, upper middle class and upper class at the 1% level. However, the tax burden has a negative statistically significant effect on the welfare attitudes of the lower class at the 5% level. This difference could be because the primary concern of the lower class is survival. Additionally, their total income may not reach the tax threshold, resulting in a weak perception of the government’s ability to balance the wealth gap. This may have contributed to the insignificant results reflected in the regression analysis. However, the tax burden has a significant effect on the welfare attitudes of other social class groups, a more reasonable tax burden can reduce their reliance on government for welfare redistribution.

**Table 8:** Heterogeneity analysis results among different social classes.

	(1)	(2)	(3)	(4)	(5)	(6)
Variable	Lower class	Working class	Lower middle class	Middle class	Upper middle class	Upper class
Tax burden	-0.107**	-0.211***	-0.304***	-0.331***	-0.508***	-0.793***
	(-2.29)	(-8.97)	(-11.42)	(-17.78)	(-14.69)	(-4.05)
Gender	-0.0602	-0.0597	-0.203***	-0.202***	-0.175***	-0.240
	(-0.53)	(-1.38)	(-4.25)	(-6.46)	(-2.99)	(-0.79)
Age	-0.00507	-0.00454***	-0.00318*	-0.00460***	-0.00636***	-0.0104
	(-1.41)	(-2.68)	(-1.76)	(-3.72)	(-2.76)	(-0.88)
Marital status	0.0736	-0.0825*	-0.0347	-0.0586*	0.00518	-0.167
	(0.58)	(-1.69)	(-0.66)	(-1.65)	(0.07)	(-0.48)
Educational attainment	0.0285	-0.0241	-0.0218	0.000470	0.0170	0.171
	(0.65)	(-1.32)	(-1.21)	(0.04)	(0.70)	(1.54)
Household size	0.0380	0.0358	0.00243	0.0246	0.0394	-0.0303
	(0.81)	(1.58)	(0.09)	(1.36)	(1.06)	(-0.14)
Number of children	-0.0586	-0.0436	0.00154	-0.0497**	-0.0701	0.0220
	(-0.86)	(-1.31)	(0.04)	(-2.06)	(-1.47)	(0.07)
Employment status	-0.0119	-0.0432	-0.100*	-0.0429	-0.102	0.0909
	(-0.09)	(-0.83)	(-1.84)	(-1.16)	(-1.46)	(0.26)
Regional fixed effects	Yes	Yes	Yes	Yes	Yes	Yes
/						
cut1	-1.867***	-2.453***	-2.890***	-2.850***	-3.048***	-4.228***
	(-5.62)	(-14.14)	(-14.73)	(-20.30)	(-11.10)	(-3.32)
cut2	-1.159***	-1.664***	-2.170***	-2.050***	-2.244***	-3.605***
	(-3.47)	(-9.65)	(-11.01)	(-14.79)	(-8.35)	(-2.89)
cut3	-0.647*	-1.103***	-1.530***	-1.442***	-1.703***	-3.231***
	(-1.91)	(-6.46)	(-7.88)	(-10.52)	(-6.40)	(-2.66)
cut4	0.260	0.0261	-0.383**	-0.206	-0.598**	-1.546
	(0.77)	(0.15)	(-2.00)	(-1.52)	(-2.27)	(-1.32)
*N*	437	2553	2093	4775	1370	73
*Pseudo R* ^2^	0.032	0.036	0.047	0.057	0.082	0.243

Note: *, **, and *** indicate statistical significance at the 10%, 5%, and 1% levels, respectively.

## 6 Mechanisms test

### 6.1 The mediating effect of perceived fairness

Combined with the previous theoretical analysis, this study argues that the effect of tax burden on welfare attitudes may be transmitted through the perception of social fairness, with the path: tax burden (taxburden) → perceived fairness (fair) → welfare attitudes (attitude). Reasonable tax burden increases residents’ perceived social fairness, which in turn decreases their dependence on and appeal to the role of the government. This study presents the concept of social fairness as a mediating variable, using the Baron and Kenny [126] stepwise method for testing the mediating effect. The mediation effect model is then combined with the benchmark regression model:

$$attitude_{i}=\alpha_{0}+\alpha_{1}taxburden_{i}+\alpha_{2}control_{i}+\mu_{i}+\varepsilon_{i}$$ (1)

$$fair_{i}=\beta_{0}+\beta_{1}taxburden_{i}+\beta_{2}control_{i}+\mu_{i}+\varepsilon_{i}$$ (2)

$$attitude_{i}=\gamma_{0}+\gamma_{1}taxburden_{i}+\gamma_{2}fair_{i}+\gamma_{3}control_{i}+\mu_{i}+\varepsilon_{i}$$ (3)

The stepwise method was used to test the regression coefficients of the core variables in the three equations. The mediating effect exists when the coefficient α_1_ is significant and both coefficients β_1_ and γ_2_ are significant. When γ_1_ is not significant, it indicates a full mediation effect. If γ_1_ is significant, and β_1_ × γ_2_ has the same sign as γ_1_, it indicates a partial mediation effect. If β_1_ × γ_2_ is different from γ_1_, it indicates a suppression (masking) effect [127].

In the mediation effect test, we used question Q16 from the questionnaire: "Do you think the income distribution in your country is fair?" The respondents had the following options: "1 = very fair," "2 = fair," "3 = unfair," and "4 = very unfair." To facilitate the interpretation of the regression results, we inversely assigned the values so that a higher value indicates that the respondents perceive a higher degree of social justice. The table below reports the results of regression analyses conducted using both OProbit and OLS methods to estimate the mediating effect of perceived fairness.

In **Table 9**, the Logit results show that when perceived fairness and tax burden are used as explanatory variables in Column (1) and Column (2), the effect of tax burden on perceived fairness is positive at the 1% level of significance. This indicates that as the reasonableness of the tax burden increases, the respondents’ perception of social fairness also increases. Comparison of the results in columns (3) and (4) with the benchmark regression results shows that the tax burden has a significantly negative effect on welfare attitudes. Additionally, perceived fairness also has a negative and significant effect on welfare attitudes at the 1% level. This suggests that individuals with a higher sense of social fairness exhibit a lower degree of dependence on government benefits.

**Table 9:** Mediating effect results from stepwise regression analysis on perceived fairness.

	(1)	(2)	(3)	(4)
Variable	Perceived fairness	Perceived fairness	Welfare attitude	Welfare attitude
	**OProbit**	**OLS**	**OProbit**	**OLS**
Tax burden	0.267***	0.151***	-0.244***	-0.233***
	(21.64)	(22.93)	(-20.62)	(-21.92)
Perceived fairness			-0.536***	-0.497***
			(-27.37)	(-29.04)
Control variable	Yes	Yes	Yes	Yes
Regional fixed effects	Yes	Yes	Yes	Yes
N	11301	11301	11301	11301
R^2^ /Pseudo R^2^	0.089	0.171	0.088	0.227

Note: *, **, and *** indicate statistical significance at the 10%, 5%, and 1% levels, respectively.

Therefore, in the stepwise regression test of the mediating effect of social equity, the coefficients β_1_, γ_1_, and γ_2_ are all significant. While β_1_ × γ_2_ and γ_1_ have the same sign, indicating that the role of perceived fairness is a partial mediating effect between tax burden and welfare attitudes. The residents’ perceived fairness is positively affected by a reasonable degree of tax burden. This means that the more reasonable the tax burden is, the higher the degree of fairness of social distribution perceived by the residents. However, perceived fairness negatively affects the attitude towards welfare. The residents believe that the social distribution is fair and that the income they receive is directly proportional to their own efforts. They do not need to resort to the government for re-adjustment. However, social fairness is a complex concept and cannot be fully explained by the tax burden alone. It is important to note that this is a partial effect and other factors may also contribute to changes in perceived fairness. Therefore, social fairness only partially mediates the relationship between the tax burden and welfare attitudes, supporting **H2** and **H3**.

The use of stepwise regression in research has been questioned by some scholars due to its low testing performance compared to other methods [128, 129]. To further test the mediation effect in the results of the hierarchical regression analysis, the Bootstrap method was used with a sampling number of 1,000. **Table 10** displays the results of the data test. The coefficient for the mediation effect of tax burden on welfare attitudes through perceived fairness is -0.0514705, which is consistent with the results of the hierarchical regression mentioned earlier. The 95% confidence interval for this coefficient ranges from -0.0595165 to -0.0434245. The direct effect coefficient of perceived fairness on welfare attitudes is -0.156103, which is consistent with the previous stepwise regression results. The 95% confidence interval is from -0.1746658 to -0.1375401. Both confidence intervals do not include 0, so it can be inferred that the perceived fairness partially mediates the relationship between tax burdens and attitudes toward welfare. This further confirms the results of the previous stepwise regression on the intermediary effect.

**Table 10:** Mediating effect test results using Bootstrap method.

	Observed	Bootstrap			Normal	based
	coefficient	std. err.	z	P>z	[95% conf.	interval]
_bs_1	-0.0514705	0.0041052	-12.54	0	-0.0595165	-0.0434245
_bs_2	-0.156103	0.009471	-16.48	0	-0.1746658	-0.1375401

### 6.2 The moderating effect of government efficacy

To assess the moderating role on the relationship between tax burdens and social welfare attitudes, we employ the following equation to analyze the impact of government efficacy:

$$attitude_{i}=\alpha_{3}+\alpha_{4}taxburden_{i}+\alpha_{5}goveffic_{i+}\alpha_{6}taxburden_{i}\times goveffic_{i+}\alpha_{7}control_{i}+\mu_{i}+\varepsilon_{i}$$ (4)

In Eq (4), the explanatory variable *attitude* is the welfare attitude of the individuals; the explanatory variable *taxburden* indicates the perceived tax burden of the individuals; *goveffic* indicates the ability and competence of a government to efficiently and effectively carry out its duties, policies, and services to meet the needs and expectations of its citizens; *taxburden×goveffic* represents the interaction between tax burden and government efficacy; *control* is a series of control variables; *ε* is the random error term; *i* represents the individuals in the sample. The sign and significance of *α*_*6*_ require our attention to analyze the moderating effect of government efficacy.

In **Table 11**, we can observe that government effectiveness negatively moderates the relationship between tax burden and welfare attitudes to some extent. Specifically, as government effectiveness increases, people’s reliance on government welfare provision decreases. This negative moderating effect may stem from changes in people’s expectations and perceptions of government performance. In an environment of high-performance government, people usually show higher satisfaction with the quality and efficiency of public services and social welfare. As a result, they are less inclined to demand further government intervention in the distribution and provision of welfare. This attitude may reflect their trust in the existing functions of the government and their empowerment. However, when people perceive the government’s effectiveness to be insufficient, especially when public services and social welfare fail to meet their expectations, they are more inclined to call on the government to increase its intervention in the welfare sector and to strengthen the provision of welfare and the regulation of its distribution. This increase in demand may be due to their expectation that the government can provide a more efficient and equitable distribution of resources. It is worth noting that the negative moderating effect of government effectiveness may vary depending on factors such as region, economic level and cultural background. For example, in more economically developed regions, people’s expectations of government effectiveness may be higher, and thus the negative effect of tax burden on welfare attitudes may be more significant. In contrast, in less economically developed regions, despite lower government efficacy, the population’s need for welfare may be more pressing, thereby weakening this negative moderating effect. From the empirical analysis above it can be concluded that government efficacy negatively moderates the impact of tax burden on welfare attitudes, confirming **H4**.

**Table 11:** Moderating effect test results.

	(1)	(2)
Variable	Welfare attitude	Welfare attitude
Tax burden	-0.278***	-0.289***
	(-26.55)	(-27.44)
Government efficacy	-0.251***	-0.245***
	(-22.35)	(-21.96)
Government efficacy×Tax burden		-0.0697***
		(-6.90)
Control variable	Yes	Yes
Regional fixed effects	Yes	Yes
N	-0.0697***	-0.0697***
R2 / Pseudo R2	(-6.90)	(-6.90)

Note: *, **, and *** indicate statistical significance at the 10%, 5%, and 1% levels, respectively.

## 7 Conclusions

### 7.1 Main findings

This study examines the intricate interplay between tax burden, perceptions of social fairness, and welfare attitudes across 11 diverse welfare countries or regions, as elucidated in the comprehensive ISSP 2019 survey. The findings reveal a crucial nexus: individuals’ perceptions of the tax burden exert a substantial influence over their attitudes towards welfare policies. It is noteworthy that residents who perceive the tax burden as reasonable tend to exhibit weaker welfare attitudes, attributing a lesser responsibility to the government for income redistribution. Conversely, those who deem the tax burden unreasonable display stronger inclinations towards welfare and tend to attribute a greater responsibility to the government for redistributive endeavors.

To fortify the credibility of these insightful findings, a meticulous robustness test was administered, wherein alternative empirical analysis methods and measures for explanatory and response variables were meticulously employed. Moreover, the study reveals a consistent negative correlation between tax burden and welfare attitudes across the four distinct welfare system categories meticulously scrutinized. However, the impact of the tax burden is nuanced and varies across social classes, with all experiencing a significantly negative effect.

Furthermore, the pivotal notion of perceived fairness emerges as a critical mediator in the complex tax burden-welfare attitude dynamic. Proposing to enhance the rationality and fairness of the tax burden represents a promising strategy for augmenting the public’s perception of social fairness, thereby tempering their expectations from government welfare provisions. This profound insight underscores the intricate relationship between fiscal policies, perceptions of fairness, and societal expectations, offering invaluable guidance for policymakers striving to institute interventions aimed at fostering a more equitable and supportive welfare system. At the same time, government efficacy plays an important negative moderating role between tax burdens and welfare attitudes, and improving government efficacy helps people to reduce their demands for government involvement in the provision and distribution of welfare.

### 7.2 Contributions and implications

Schumpeter contends that the essence of modern states lies in their capacity to levy taxes, a cornerstone of their sustenance. The ability to draw taxes is pivotal in reconciling the tension between the tax burden and the welfare objectives of the state, thereby delineating the extent of social welfare provision and even determining the welfare state’s survival. Tax reforms within welfare states have historically vacillated between increments and reductions, driven by the dual imperative of bolstering revenue streams to meet burgeoning public service demands and garnering public support. Yet, both strategies entail inherent challenges. Heightened taxation exacerbates an already burdensome tax load, eliciting resistance, while tax cuts diminish governmental fiscal reservoirs, jeopardizing the maintenance of established welfare standards. Neither unilateral tax hikes nor reductions suffice to yield optimal outcomes.

The findings presented herein underscore the salience of a judicious tax burden in fostering societal perceptions of social equity while alleviating pressure on the state to fulfill welfare obligations. Given the entrenched rigidity within welfare structures, tax reforms within such contexts necessitate the preservation or marginal augmentation of total tax revenue without exacerbating public perception of the tax burden. This epitomizes the precept of "taxation within capacity," wherein individuals of commensurate tax capacity contribute equitably while ensuring that the tax burden remains within the economic and psychological realms of societal affordability. This proposition strives to uphold a tax regime consonant with the majority’s economic and psychological thresholds, thereby enhancing perceptions of social justice in income distribution regulation through taxation.

Notably, tax structures vary across nations, with the United Kingdom and the United States predominantly relying on direct and indirect taxes, while Germany and France exhibit a contrasting preference. These configurations are calibrated to effect a moderate expansion of total tax revenue without perceptibly heightening the tax burden. Most Western democracies have embraced a tax regime predicated on direct taxation conducive to representative governance. Conversely, centralized systems often favor indirect levies like value-added tax (VAT) or goods and services taxes. It is imperative to align tax structures with prevailing governance systems, as evident in instances of mismatch, where adopting indirect taxation within democratic frameworks or vice versa precipitates operational inefficiencies or societal disarray.

Sustaining a stable tax framework curtails compliance costs and fosters predictability in tax obligations, thereby optimizing societal welfare. Insights gleaned from welfare state research furnish valuable guidance for constructing social welfare systems in developing countries. Many such nations grapple with escalating income disparities, impeding economic and societal harmony. To redress this imbalance, governments must regulate income distribution through taxation and allied measures. Given the infeasibility of establishing full-fledged welfare states due to structural constraints, developing nations should instead endeavor to craft modern, sustainable tax and welfare systems commensurate with their developmental trajectories. Policies prioritizing equitable opportunities, narrowing income differentials, and ensuring universal access to basic public services hold promise in enhancing societal inclusivity and cohesion.

### 7.3 Limitations and future research

Future research endeavors should address several limitations identified in this study, which will contribute to advancing the empirical understanding of welfare attitudes and tax burdens:

Firstly, there is a need to enhance sample representation. While the utilization of data exclusively from the ISSP 2019 survey offers valuable insights, the risk of sample selection bias cannot be ignored. To mitigate this concern, future research should broaden the scope of sampling to encompass a more diverse array of welfare states. By incorporating data from various regions and socio-economic contexts, researchers can improve the representativeness and robustness of their analyses.

Secondly, advancements in measurement methodologies are essential. The subjective nature of measuring welfare attitudes and tax burdens presents inherent challenges, potentially leading to measurement errors and compromising the validity of study outcomes. To address this limitation, future investigations should explore innovative measurement methodologies that minimize subjectivity and ensure precise operationalization of variables. Incorporating objective data sources alongside subjective assessments can further enhance the accuracy and reliability of findings. By adopting more rigorous measurement approaches grounded in objective data, researchers can strengthen the empirical foundation of their analyses and deepen our understanding of welfare attitudes and tax burdens.

Lastly, longitudinal analysis for causal inference is imperative. The cross-sectional design of the study poses constraints on establishing causal relationships between tax burdens and welfare attitudes. While the OProbit model provides insights into the impact of tax burden on welfare attitudes, it falls short of elucidating causal pathways. To overcome this limitation, future research endeavors could leverage panel data to longitudinally track individuals’ or households’ welfare attitudes and tax burdens. By examining changes and trends over time, researchers can gain a deeper understanding of the causal relationship between tax burden and welfare attitudes, thereby advancing knowledge in this field.

## References

[1] Midgley JO. Social development: The developmental perspective in social welfare. Social Development. 1995; 1–208.

[2] Woolcock M. Social capital and economic development: Toward a theoretical synthesis and policy framework. Theory and society. 1998;27: 151–208.

[3] Roseland M. Sustainable community development: integrating environmental, economic, and social objectives. Progress in planning. 2000;54: 73–132.

[4] Rothstein B, Uslaner EM. All for all: Equality, corruption, and social trust. World politics. 2005;58: 41–72.

[5] Malhotra N. Role of Government to Ensure Economic and Social Welfare. Microfinance and Development in Emerging Economies: An Alternative Financial Model for Advancing the SDGs. Emerald Publishing Limited; 2023. pp. 161–188. Available: https://www.emerald.com/insight/content/doi/10.1108/978-1-83753-826-320231008/full/html

[6] Esping-Andersen G. The three worlds of welfare capitalism. Princeton University Press; 1990. Available: https://books.google.com/books?hl=zh-CN&lr=&id=ERCnDwAAQBAJ&oi=fnd&pg=PR8&dq=Three+Worlds+of+Welfare+Capitalism&ots=0EbrhJb1Oq&sig=3GZvzvDD3sd8u-WrVaFXZkyyNUo

[7] Brooks C, Manza J. Why Do Welfare States Persist? The Journal of Politics. 2006;68: 816–827. doi: 10.1111/j.1468-2508.2006.00472.x

[8] Ringen S. The possibility of politics: A study in the political economy of the welfare state. Routledge; 2017. Available: https://www.taylorfrancis.com/books/mono/10.4324/9781315134062/possibility-politics-stein-ringen

[9] Mäntyneva P. Do welfare regimes matter? Perceptions of welfare in contemporary word. International Journal of Sociology and Social Policy. 2023 [cited 27 Jan 2024]. Available: https://www.emerald.com/insight/content/doi/10.1108/IJSSP-09-2023-0236/full/html

[10] Pierson P. Coping with permanent austerity: welfare state restructuring in affluent democracies. Revue française de sociologie. 2002; 369–406.

[11] Glennerster H. The sustainability of western welfare states. 2010 [cited 20 Feb 2024]. Available: https://academic.oup.com/edited-volume/34386/chapter/291617306

[12] Dole JA, Sinatra GM. Social psychology research on beliefs and attitudes: Implications for research on learning from text. Beliefs about text and instruction with text. Routledge; 2019. pp. 245–264. Available: https://www.taylorfrancis.com/chapters/edit/10.4324/9780203812068-13/social-psychology-research-beliefs-attitudes-implications-research-learning-text-janice-dole-gale-sinatra

[13] Lönnqvist J-E, Kivikangas M. Economic attitudes, social attitudes and their psychological underpinnings–A study of the Finnish political elite. Frontiers in psychology. 2019;10: 602. doi: 10.3389/fpsyg.2019.00602 30941081 PMC6433877

[14] Attewell D. Deservingness perceptions, welfare state support and vote choice in Western Europe. West European Politics. 2021;44: 611–634. doi: 10.1080/01402382.2020.1715704

[15] Choi G. Individuals’ socioeconomic position, inequality perceptions, and redistributive preferences in OECD countries. J Econ Inequal. 2021;19: 239–264. doi: 10.1007/s10888-020-09471-6

[16] Lucas GM Jr. The Pain of Paying Taxes. U Rich L Rev. 2021;56: 545.

[17] Arikan G, Bloom B-N. Social Values and Cross-National Differences in Attitudes towards Welfare. Political Studies. 2015;63: 431–448. doi: 10.1111/1467-9248.12100

[18] Gelepithis M, Giani M. Inclusion without Solidarity: Education, Economic Security, and Attitudes toward Redistribution. Political Studies. 2022;70: 45–61. doi: 10.1177/0032321720933082

[19] Andreß H-J, Heien T. Four worlds of welfare state attitudes? A comparison of Germany, Norway, and the United States. European Sociological Review. 2001;17: 337–356.

[20] Arts W, Gelissen J. Welfare States, Solidarity and Justice Principles: Does the Type Really Matter? Acta Sociologica. 2001;44: 283–299. doi: 10.1177/000169930104400401

[21] Shorrocks R, Grasso MT. The attitudinal gender gap across generations: Support for redistribution and government spending in contexts of high and low welfare provision. European Political Science Review. 2020;12: 289–306.

[22] Robinson RV, Bell W. Equality, success, and social justice in England and the United States. American Sociological Review. 1978; 125–143.

[23] Svallfors S. Class, Attitudes and the Welfare State: Sweden in Comparative Perspective. Soc Policy Adm. 2004;38: 119–138. doi: 10.1111/j.1467-9515.2004.00381.x

[24] Gryaznova O. Factors affecting welfare attitudes in different types of welfare states: Personal interests and values. Higher School of Economics Research Paper No WP BRP. 2013;18. Available: https://papers.ssrn.com/sol3/papers.cfm?abstract_id=2267337

[25] Taylor-Gooby P. Attitudes to welfare. Journal of Social Policy. 1985;14: 73–81.

[26] Baute S, Meuleman B. Public attitudes towards a European minimum income benefit: How (perceived) welfare state performance and expectations shape popular support. Journal of European Social Policy. 2020;30: 404–420. doi: 10.1177/0958928720904320

[27] Svallfors S. Worlds of welfare and attitudes to redistribution: A comparison of eight western nations. European sociological review. 1997;13: 283–304.

[28] Hasenfeld Y, Rafferty JA. The determinants of public attitudes toward the welfare state. Social Forces. 1989;67: 1027–1048.

[29] Blomberg H, Kroll C. Do Structural Contexts Matter? Macro-sociological Factors and Popular Attitudes towards Public Welfare Services. Acta Sociologica. 1999;42: 319–335. doi: 10.1177/000169939904200403

[30] Blekesaune M, Quadagno J. Public attitudes toward welfare state policies: A comparative analysis of 24 nations. European sociological review. 2003;19: 415–427.

[31] Roosma F, Gelissen J, Van Oorschot W. The multidimensionality of welfare state attitudes: A European cross-national study. Social indicators research. 2013;113: 235–255. doi: 10.1007/s11205-012-0099-4 23874057 PMC3696173

[32] Borg J, Gustafsson C, Landerdahl Stridsberg S, Zander V. Implementation of welfare technology: a state-of-the-art review of knowledge gaps and research needs. Disability and Rehabilitation: Assistive Technology. 2023;18: 227–239. doi: 10.1080/17483107.2022.2120104 36103349

[33] Gründler K, Köllner S. Culture, diversity, and the welfare state. Journal of Comparative Economics. 2020;48: 913–932.

[34] Jaeger MM. United but divided: Welfare regimes and the level and variance in public support for redistribution. European Sociological Review. 2009;25: 723–737.

[35] Pfau-Effinger B. Culture and welfare state policies: Reflections on a complex interrelation. Journal of social policy. 2005;34: 3–20.

[36] Baranowski M, Jabkowski P. How many worlds of welfare state attitudes are there? European experiences in a comparative perspective of cross-national survey research. Economics & Sociology. 2022;15: 160–177.

[37] Inglehart R. The renaissance of political culture. American political science review. 1988;82: 1203–1230.

[38] Inglehart R, Baker WE. Modernization, cultural change, and the persistence of traditional values. American sociological review. 2000; 19–51.

[39] Kafka KI, Kostis PC. Post-materialism and economic growth: Cultural backlash, 1981–2019. Journal of Comparative Economics. 2021;49: 901–917.

[40] Whyte MK, Han C. Popular Attitudes toward Distributive Injustice: Beijing and Warsaw Compared. J OF CHIN POLIT SCI. 2008;13: 29–51. doi: 10.1007/s11366-008-9016-8

[41] He AJ, Qian J, Ratigan K. Attitudes toward welfare spending in urban China: Evidence from a survey in two provinces and social policy implications. Journal of Chinese Governance. 2021;6: 131–154.

[42] Yeh C-Y, Ku Y-W. Welfare attitude and economic developmentalism in new democratic developmental welfare state: an examination of the Taiwanese case. Journal of Asian Public Policy. 2021;14: 13–29.

[43] Beramendi P, Rehm P. Who Gives, Who Gains? Progressivity and Preferences. Comparative Political Studies. 2016;49: 529–563. doi: 10.1177/0010414015617961

[44] Okafor OG, Igbinovia MI. Taxation: A tool for environmental conservation. International Journal of Economics, Commerce and Management. 2017;5: 68–81.

[45] Egiyi MA. Taxation as a Significant Tool for Economic Development. International Journal of Economics and Public Policy. 2022;6: 12–22.

[46] Ozai I. Two accounts of international tax justice. Canadian Journal of Law & Jurisprudence. 2020;33: 317–339.

[47] Croce M, Nguyen TT, Raymond S. Persistent government debt and aggregate risk distribution. Journal of Financial Economics. 2021;140: 347–367.

[48] Wilkinson BR, Hageman AM. The role of political elites in income tax system design and tax fairness. The British Accounting Review. 2023;55: 101172.

[49] Miller AH, Borrelli SA. Confidence in Government During the 1980s. American Politics Quarterly. 1991;19: 147–173. doi: 10.1177/1532673X9101900201

[50] Stanley L. Legitimacy gaps, taxpayer conflict, and the politics of austerity in the UK. The British Journal of Politics and International Relations. 2016;18: 389–406. doi: 10.1177/1369148115615031

[51] Avi-Yonah RS. Globalization, tax competition, and the fiscal crisis of the welfare state. Harvard law review. 2000; 1573–1676.

[52] Murshed SM, Tadjoeddin MZ. Revisiting the greed and grievance explanations for violent internal conflict. J of Intl Development. 2009;21: 87–111. doi: 10.1002/jid.1478

[53] Fjeldstad O-H, Schulz-Herzenberg C, Hoem Sjursen I. People’s views of taxation in Africa: a review of research on determinants of tax compliance. Available at SSRN 2411424. 2012 [cited 28 Jan 2024]. Available: https://papers.ssrn.com/sol3/papers.cfm?abstract_id=2411424

[54] Saruji SC, Mohdali R, Mohamed NNN. Trust in government and perceptions of tax compliance among adolescents. Journal of Advanced Research Design. 2019;61: 19–29.

[55] Widuri R, Irawan W. Tax justice perception and trust in government on tax compliance. International Conference on Tourism, Economics, Accounting, Management, and Social Science (TEAMS 19). Atlantis Press; 2019. pp. 105–110. Available: https://www.atlantis-press.com/proceedings/teams-19/125924368

[56] Tambun S, Haryati A. The Effect of Satisfaction on Public Services, Trust in Government and Perception of Corruption on Tax Awareness through Tax Morals. Integrated Journal of Business and Economics. 2022;6: 74–86.

[57] Atrostic BK, Nunns JR. Measuring tax burden: A Historical perspective. Fifty years of economic measurement: The jubilee of the conference on research in income and wealth. University of Chicago Press; 1991. pp. 343–420. Available: https://www.nber.org/system/files/chapters/c5981/c5981.pdf

[58] Celikay F. Dimensions of tax burden: a review on OECD countries. Journal of Economics, Finance and Administrative Science. 2020;25: 27–44.

[59] Karni E, Salmon T, Sopher B. Individual sense of fairness: an experimental study. Exp Econ. 2008;11: 174–189. doi: 10.1007/s10683-007-9165-1

[60] Lindahl E. Die Gerechtigkeit der Besteuerung. Eine Analyse der Steuerprinzipien auf der Grundlage der Grenznutzentheorie, Håkan Ohlssons Buchdruckerei, Lund, partially translated as “Just Taxation: A Positive Solution.” RA Musgrave and AT Peacock, Classics in the Theory of Public Finance, MacMillan, New York. 1919; 168–176.

[61] Benabou R. Unequal societies: Income distribution and the social contract. American Economic Review. 2000;91: 96–129.

[62] Uler N. Public goods provision and redistributive taxation. Journal of Public Economics. 2009;93: 440–453.

[63] Ostrom V, Ostrom E. Public goods and public choices. Alternatives for delivering public services. Routledge; 2019. pp. 7–49. Available: https://www.taylorfrancis.com/chapters/edit/10.4324/9780429047978-2/public-goods-public-choices-vincent-ostrom-elinor-ostrom

[64] Yamagishi T. The provision of a sanctioning system as a public good. Journal of Personality and social Psychology. 1986;51: 110.

[65] Potts J. The innovation deficit in public services: The curious problem of too much efficiency and not enough waste and failure. Innovation. 2009;11: 34–43. doi: 10.5172/impp.453.11.1.34

[66] Wagner RE. Revenue structure, fiscal illusion, and budgetary choice. Public choice. 1976; 45–61.

[67] Logan RR. Fiscal Illusion and the Grantor Government. Journal of Political Economy. 1986;94: 1304–1318. doi: 10.1086/261434

[68] Dollery BE, Worthington AC. THE EMPIRICAL ANALYSIS OF FISCAL ILLUSION. Journal of Economic Surveys. 1996;10: 261–297. doi: 10.1111/j.1467-6419.1996.tb00014.x

[69] Wildowicz-Giegiel A, Kargol-Wasiluk A. The phenomenon of fiscal illusion from theoretical and empirical perspective: the case of Euro Area countries. 2020 [cited 28 Jan 2024]. Available: https://www.um.edu.mt/library/oar/handle/123456789/57583

[70] Weisstanner D, Armingeon K. Redistributive preferences: Why actual income is ultimately more important than perceived income. Journal of European Social Policy. 2022;32: 135–147. doi: 10.1177/09589287211037912

[71] Giuliodori M, Parlevliet J, Rooduijn M. Populist attitudes, fiscal illusion and fiscal preferences: evidence from Dutch households1. Fiscal Illusion and Fiscal Preferences: Evidence from Dutch Households1. 2022 [cited 28 Jan 2024]. Available: https://papers.ssrn.com/sol3/papers.cfm?abstract_id=4232307

[72] Kirk G, Fernandes A, Friedman B. Who Pays for the Welfare State? Austerity Politics and the Origin of Pay-to-Stay Fees as Revenue Generation. Sociological Perspectives. 2020;63: 921–938. doi: 10.1177/0731121420967037

[73] Coote A. Towards a sustainable welfare state: the role of universal basic services. Social Policy and Society. 2022;21: 473–483.

[74] Yang K, Peng H, Chen J. Chinese seniors’ attitudes towards government responsibility for social welfare: Self-interest, collectivism orientation and regional disparities. International Journal of Social Welfare. 2019;28: 208–216.

[75] Caldwell S, Nelson S, Waldinger D. Tax Refund Uncertainty: Evidence and Welfare Implications. American Economic Journal: Applied Economics. 2023;15: 352–376. doi: 10.1257/app.20210383

[76] Mussweiler T. Comparison processes in social judgment: mechanisms and consequences. Psychological review. 2003;110: 472. doi: 10.1037/0033-295x.110.3.472 12885111

[77] Galesic M, Olsson H, Rieskamp J. A sampling model of social judgment. Psychological review. 2018;125: 363. doi: 10.1037/rev0000096 29733664

[78] Rawls J. Atheory of justice. Cambridge (Mass). 1971 [cited 28 Jan 2024]. Available: https://books.google.com/books?hl=zh-CN&lr=&id=3M0xHAVZQnUC&oi=fnd&pg=PA387&dq=justice+is+the+primary+value+of+the+social+system+rawls&ots=Pf7lrmJyuw&sig=hy3P8XyKAPGRj-xLIXopwleaD-I

[79] Frederickson G. The state of social equity in American public administration. National Civic Review. 2005;94: 31–38.

[80] Lewin-Epstein N, Kaplan A, Levanon A. Distributive justice and attitudes toward the welfare state. Social Justice Research. 2003;16: 1–27. doi: 10.1023/A:1022909726114

[81] Sabbagh C, Vanhuysse P. Exploring attitudes towards the welfare state: Students’ views in eight democracies. Journal of Social Policy. 2006;35: 607–628.

[82] Calzada I, Gómez-Garrido M, Moreno L, Moreno-Fuentes FJ. It is not only about equality. A study on the (other) values that ground attitudes to the welfare state. International Journal of Public Opinion Research. 2014;26: 178–201.

[83] Findor A. Moral Foundations of Welfare Attitudes: The Role of Moral Intuition and Reasoning in Pursuing Social Justice. Central European Journal of Public Policy. 2015;9: 72–83. doi: 10.1515/cejpp-2016-0013

[84] Sachweh P. Conditional Solidarity: Social Class, Experiences of the Economic Crisis, and Welfare Attitudes in Europe. Soc Indic Res. 2018;139: 47–76. doi: 10.1007/s11205-017-1705-2

[85] Van Hootegem A, Abts K, Meuleman B. Differentiated Distributive Justice Preferences? Configurations of Preferences for Equality, Equity and Need in Three Welfare Domains. Soc Just Res. 2020;33: 257–283. doi: 10.1007/s11211-020-00354-9

[86] Ratzmann N. Deserving of social support? Street-level bureaucrats’ decisions on EU migrants’ benefit claims in Germany. Social Policy and Society. 2021;20: 509–520.

[87] Koch M. Social policy without growth: Moving towards sustainable welfare states. Social Policy and Society. 2022;21: 447–459.

[88] Schakel W, Burgoon B, Hakhverdian A. Real but Unequal Representation in Welfare State Reform. Politics & Society. 2020;48: 131–163. doi: 10.1177/0032329219897984

[89] Krawczyk M. A glimpse through the veil of ignorance: Equality of opportunity and support for redistribution. Journal of Public Economics. 2010;94: 131–141.

[90] Berkman ET, Livingston JL, Kahn LE. Finding The “Self” in Self-Regulation: The Identity-Value Model. Psychological Inquiry. 2017;28: 77–98. doi: 10.1080/1047840X.2017.1323463 30774280 PMC6377081

[91] Condon M, Wichowsky A. Inequality in the Social Mind: Social Comparison and Support for Redistribution. The Journal of Politics. 2020;82: 149–161. doi: 10.1086/705686

[92] Piketty T. Social mobility and redistributive politics. The Quarterly journal of economics. 1995;110: 551–584.

[93] Dornstein M. Taxes: Attitudes and perceptions and their social bases. Journal of Economic Psychology. 1987;8: 55–76.

[94] Kinsey KA, Grasmick HG, Smith KW. Framing justice: Taxpayer evaluations of personal tax burdens. Law and Society Review. 1991; 845–873.

[95] Cuccia AD, Carnes GA. A closer look at the relation between tax complexity and tax equity perceptions. Journal of Economic Psychology. 2001;22: 113–140.

[96] Alexander P, Balavac-Orlic M. Tax morale: Framing and fairness. Economic Systems. 2022;46: 100936.

[97] Roosma F, Van Oorschot W, Gelissen J. A just distribution of burdens? Attitudes toward the social distribution of taxes in 26 welfare states. International Journal of Public Opinion Research. 2016;28: 376–400.

[98] Fleurbaey M, Maniquet F. Optimal income taxation theory and principles of fairness. Journal of Economic Literature. 2018;56: 1029–1079.

[99] Saez E, Stantcheva S. Generalized social marginal welfare weights for optimal tax theory. American Economic Review. 2016;106: 24–45.

[100] Hvidberg KB, Kreiner CT, Stantcheva S. Social positions and fairness views on inequality. Review of Economic Studies. 2023;90: 3083–3118.

[101] Farrar J, Massey DW, Osecki E, Thorne L. Tax Fairness: Conceptual Foundations and Empirical Measurement. J Bus Ethics. 2020;162: 487–503. doi: 10.1007/s10551-018-4001-4

[102] Schmölders G. Das Irrationale in der öffentlichen Finanzwirtschaft [The irrational in public finance]. Frankfurt am Main: Suhrkamp. 1960.

[103] Pukeliene V, Kažemekaityte A. Tax behaviour: assessment of tax compliance in European Union countries. Ekonomika. 2016;95: 30–56.

[104] Febrian YB, Islami IN. Gender, trust, and tax compliance: the mediating effect of fairness perception. JAAF (Journal of Applied Accounting and Finance). 2020;4: 131–145.

[105] Miller G. Above politics: Credible commitment and efficiency in the design of public agencies. Journal of Public Administration Research and Theory. 2000;10: 289–328.

[106] Schuck PH. Why government fails so often: And how it can do better. 2014 [cited 21 Jul 2024]. Available: https://www.torrossa.com/gs/resourceProxy?an=5580319&publisher=FZO137

[107] McCaffery EJ, Baron J. Thinking about tax. Psychology, public policy, and law. 2006;12: 106.

[108] Alshawi EJ, Al-Tamimi SA, Anssari MAA, Hanoon MF. The Effect Of Applying The Principles Of Tax Governance In Increasing The Efficiency Of Tax Administration And Increase Tax Compliance. International Journal of eBusiness and eGovernment Studies. 2023;15: 68–88.

[109] Gebrihet HG, Gebresilassie YH, Woldu GT. Trust, Corruption, and Tax Compliance in Fragile States: On a Quest for Transforming Africa into Future Global Powerhouse. Social Sciences. 2023;13: 3.

[110] Khaltar O. Tax evasion and governance quality: The moderating role of adopting open government. International Review of Administrative Sciences. 2024;90: 276–294. doi: 10.1177/00208523231197317

[111] Jordan J. Policy feedback and support for the welfare state. Journal of European Social Policy, 2013, 23(2): 134–148.

[112] Bastiaens I, Beesley C. Economic Hardship and Welfare Policy Preferences: What Can the COVID-19 Pandemic Tell Us?. Political Studies Review, 2024: 14789299241252386.

[113] Sundberg T, Taylor-Gooby P. A systematic review of comparative studies of attitudes to social policy. Evidence and Evaluation in Social Policy. 2014; 63–80.

[114] ISSP Research Group (2022). International Social Survey Programme: Social Inequality V—ISSP 2019. GESIS, Köln. ZA7600 Datenfile Version 3.0.0, 10.4232/1.14009.

[115] Daniele G, Geys B. Interpersonal trust and welfare state support. European Journal of Political Economy, 2015, 39: 1–12.

[116] Barth E, Finseraas H, Kjelsrud A, et al. Openness and the welfare state: risk and income effects in protection without protectionism[J]. European Journal of Political Economy, 2023, 79: 102405.

[117] Jæger MM. Welfare regimes and attitudes towards redistribution: The regime hypothesis revisited. European Sociological Review. 2006;22: 157–170.

[118] Jakobsen TG. Welfare Attitudes and Social Expenditure: Do Regimes Shape Public Opinion? Soc Indic Res. 2011;101: 323–340. doi: 10.1007/s11205-010-9666-8

[119] Alexopoulou S, Åström J, Karlsson M. The grey digital divide and welfare state regimes: a comparative study of European countries. Information Technology & People. 2022;35: 273–291.

[120] Larsen CA. The institutional logic of welfare attitudes: How welfare regimes influence public support. Routledge; 2016. Available: https://www.taylorfrancis.com/books/mono/10.4324/9781315556727/institutional-logic-welfare-attitudes-christian-albrekt-larsen

[121] Linos K, West M. Self-interest, social beliefs, and attitudes to redistribution. Re-addressing the issue of cross-national variation. European Sociological Review. 2003;19: 393–409.

[122] D GILLIAM F, Whitby KJ. Race, class, and attitudes toward social welfare spending: An ethclass interpretation. Social Science Quarterly. 1989;70: 88.

[123] Wong TK, Wan SP, Law KW. Welfare attitudes and social class: the case of Hong Kong in comparative perspective. Int J Soc Welfare. 2009;18: 142–152. doi: 10.1111/j.1468-2397.2008.00576.x

[124] Guo J, Hao Y, Ling W. The effects of social class on social welfare attitudes A mediating role of fairness perceptions——Evidence from two welfare states and China. International Review of Economics & Finance. 2024;89: 1529–1538.

[125] Gelissen J. Popular support for institutionalised solidarity: a comparison between European welfare states. Int J Soc Welfare. 2000;9: 285–300. doi: 10.1111/1468-2397.00140

[126] Baron RM, Kenny DA. The moderator–mediator variable distinction in social psychological research: Conceptual, strategic, and statistical considerations. Journal of personality and social psychology. 1986;51: 1173. doi: 10.1037//0022-3514.51.6.1173 3806354

[127] MacKinnon DP. Equivalence of the mediation, confounding and suppression effect. Prevention Science. 2000;1: 173–181. doi: 10.1023/a:1026595011371 11523746 PMC2819361

[128] MacKinnon DP, Lockwood CM, Hoffman JM, West SG, Sheets V. A comparison of methods to test mediation and other intervening variable effects. Psychological methods. 2002;7: 83. doi: 10.1037/1082-989x.7.1.83 11928892 PMC2819363

[129] Fritz MS, MacKinnon DP. Required Sample Size to Detect the Mediated Effect. Psychol Sci. 2007;18: 233–239. doi: 10.1111/j.1467-9280.2007.01882.x 17444920 PMC2843527

