# Correction: The impact of tax burden on welfare attitudes: Micro evidence from welfare states

**DOI:** 10.1371/journal.pone.0321230

**Published:** 2025-03-27

**Authors:** Yinan Guo, Ting Gong

There are errors in the Tables 3,4 and 11. In Table 3, the values for “R^2^/ Pseudo R^2^” under columns “(3)” and “(4)” should be corrected to “0.044” and “0.054”, respectively. In Table 4, the values for “N” and “R^2^/ Pseudo R^2^” in columns “(1)”, “(2)”, “(3)”, and “(4)” are incorrect. In Table 11, the values for “N” and “R2/ Pseudo R2” in columns “(1)” and “(2)” are incorrect. Please see the correct Tables 3,4 and 11 here.

**Table 3:** Robustness test results using OLS and OLogit models.

	(1)	(2)	(3)	(4)
**Variable**	**OLS**	**OLS**	**OLogit**	**OLogit**
Tax burden	-0.317^***^	-0.307^***^	-0.552^***^	-0.543^***^
	(-29.33)	(-28.73)	(-26.81)	(-26.20)
Gender		-0.178^***^		-0.281^***^
		(-8.91)		(-7.98)
Age		-0.00465^***^		-0.00790^***^
		(-6.13)		(-5.85)
Marital status		-0.0513^**^		-0.0880^**^
		(-2.25)		(-2.21)
Education level		-0.00587		-0.00618
		(-0.77)		(-0.46)
Family size		0.0242^**^		0.0406^**^
		(2.17)		(2.05)
Number of children		-0.0389^**^		-0.0769^***^
		(-2.50)		(-2.83)
Social class		-0.113^***^		-0.204^***^
		(-10.64)		(-10.69)
Employment status		-0.0705^***^		-0.119^***^
		(-3.05)		(-2.90)
Regional fixed effects	Yes	Yes	Yes	Yes
_cons	4.356^***^	5.012^***^		
	(112.73)	(63.51)		
/				
cut1			-4.237^***^	-5.434^***^
			(-53.84)	(-36.70)
cut2			-2.834^***^	-4.009^***^
			(-37.48)	(-27.44)
cut3			-1.868^***^	-3.024^***^
			(-26.12)	(-21.04)
cut4			0.0176	-1.105^***^
			(0.26)	(-7.85)
N	11301	11301	11301	11301
R^2^/ Pseudo R^2^	0.126	0.151	0.044	0.054

Note:

*  ,

**, and

*** indicate statistical significance at the 10%, 5%, and 1% levels, respectively.

**Table 4:** Robustness test results with alternative explained variables.

	(1)	(2)	(3)	(4)
**Variable**	**Probit**	**Probit**	**Logit**	**Logit**
Tax burden 2	-0.194^***^	-0.198^***^	-0.322^***^	-0.328^***^
	(-15.04)	(-15.07)	(-14.99)	(-14.96)
Control variable	No	Yes	No	Yes
_cons	0.419^***^	0.903^***^	0.698^***^	1.476^***^
	(9.14)	(9.02)	(9.34)	(9.03)
N	10313	10313	10313	10313
Pseudo R^2^	0.061	0.072	0.061	0.072

Note:

*  ,

**, and

*** indicate statistical significance at the 10%, 5%, and 1% levels, respectively.

**Table 11:** Moderating effect test results.

	(1)	(2)
**Variable**	**Welfare attitude**	**Welfare attitude**
Tax burden	-0.278^***^	-0.289^***^
	(-26.55)	(-27.44)
Government efficacy	-0.251^***^	-0.245^***^
	(-22.35)	(-21.96)
Government efficacy×Tax burden		-0.0697^***^
		(-6.90)
Control variable	Yes	Yes
Regional fixed effects	Yes	Yes
N	11301	11301
R^2^	0.193	0.197

Note:

*,

**, and

*** indicate statistical significance at the 10%, 5%, and 1% levels, respectively.

In the Benchmark regression subsection of Results, there is an error in the first sentence of the fourth paragraph. The correct sentence is: Regarding the family characteristics factors of the control variables, it was found that family size had a positive and significant effect and the number of children showed a negative and significant effect.

In The mediating effect of perceived fairness subsection of Mechanisms test, there is an error in the first sentence of the fourth paragraph. The correct sentence is: In Table 9, the results show that when perceived fairness and tax burden are used as explanatory variables in Column (1) and Column (2), the effect of tax burden on perceived fairness is positive at the 1% level of significance.
